# Mitochondrial E3 ligase March5 maintains stemness of mouse ES cells via suppression of ERK signalling

**DOI:** 10.1038/ncomms8112

**Published:** 2015-06-02

**Authors:** Hao Gu, Qidong Li, Shan Huang, Weiguang Lu, Fangyuan Cheng, Ping Gao, Chen Wang, Lin Miao, Yide Mei, Mian Wu

**Affiliations:** 1 CAS Key Laboratory of Innate Immunity and Chronic Disease, Innovation Center for Cell Signaling Network, School of Life Sciences and Medical Center, University of Science and Technology of China, Hefei, Anhui 230027, China; 2 Pathology Department, The Second Hospital of Anhui Medical University, Hefei, Anhui 230061, China; 3 State Key Laboratory of Cell Biology, Institute of Biochemistry and Cell Biology, Shanghai Institutes for Biological Sciences, Chinese Academy of Sciences, Shanghai 200031, China; 4 Scientific and Educational Department, The Second Hospital of Anhui Medical University, Hefei, Anhui 230061, China

## Abstract

Embryonic stem cells (ESCs) possess pluripotency, which is the capacity of cells to differentiate into all lineages of the mature organism. Increasing evidence suggests that the pluripotent state of ESCs is regulated by a combination of extrinsic and intrinsic factors. The underlying mechanisms, however, are not completely understood. Here, we show that March5, an E3 ubiquitin ligase, is involved in maintaining mouse-ESC (mESC) pluripotency. Knockdown of March5 in mESCs led to differentiation from naive pluripotency. Mechanistically, as a transcriptional target of Klf4, March5 catalyses K63-linked polyubiquitination of Prkar1a, a negative regulatory subunit of PKA, to activate PKA, thereby inhibiting the Raf/MEK/ERK pathway. Moreover, March5 is able to replace a MEK/ERK inhibitor to maintain mESC pluripotency under serum-free culture conditions. In addition, March5 can partially replace the use of Klf4 for somatic cell reprogramming. Collectively, our study uncovers a role for the Klf4–March5–PKA–ERK pathway in maintaining the stemness properties of mESCs.

## Abstract (web summary)

The pluripotent state of mouse embryonic stem cells (mESCs) is regulated by extrinsic and intrinsic signals but the underlying mechanisms are not completely understood. Here the authors show that the E3 ligase, March5, contributes to the maintenance of the pluripotent state in mESCs via suppression of ERK activation.

## Graphical abstract

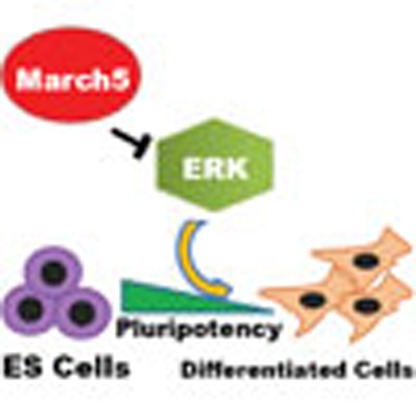

Embryonic stem cells (ESCs) are derived from the inner cell mass of the blastocyst and can be maintained in a self-renewal state while retaining the capacity for multi-lineage specification and differentiation^1^,^2^,^3^. The potential of ESCs to differentiate into specific cell types is widely used for studies of developmental processes and cell-based therapies. Therefore, to harness the full potential of ESCs, a better understanding of the molecular mechanisms underlying the regulation of ESC pluripotency is essential.

Previous studies have revealed that the pluripotency of mouse ESCs (mESCs) is maintained by multiple soluble factors, such as leukaemia inhibitory factor (LIF)^4^,^5^, bone morphogenetic protein^6^ and Wnt^7^,^8^, as well as certain nuclear transcription factors, including Stat3, Oct4 (Pou5f1), Sox2, Nanog and Kruppel-like factor 4 (Klf4)^9^. Thus, the most commonly used growth condition for mESCs is culture medium supplemented with serum and LIF, which can promote ESC self-renewal by activation of Stat3^10^,^11^. Oct4 is a critical transcription factor required to maintain an undifferentiated state and pluripotency of ESCs. This requirement is highlighted by the findings that Oct4 knockout mice are embryonically lethal and that inactivation of Oct4 in ESCs triggers conversion predominantly into trophoblast-like cells^12^. In addition to Oct4, Sox2 and Nanog are also considered to be core elements of the ESC pluripotent transcriptional network. Sox2-null embryos form normal blastocysts but fail to develop at the stage of gastrulation^13^. Nanog is essential for formation of the epiblast in the embryo^14^,^15^, and Nanog-null ESCs are highly prone to differentiation^16^. Intriguingly, Oct4, Sox2 and Nanog have been shown to co-occupy a substantial portion of their target genes, many of which are pluripotency-related genes^9^,^17^. Additionally, these three transcription factors are able to regulate their own and each other's expression in a highly coordinated manner^18^. These findings suggest that Oct4, Sox2 and Nanog form an interconnected auto-regulatory network to maintain the identity of ESCs.

A search for transcription factors downstream of LIF signalling has suggested an important role of Klf4 in regulating ESC pluripotency. Klf4 belongs to the Klf family of conserved zinc finger transcription factors. It has been shown that Klf4 is a direct target of Stat3, and overexpression of Klf4 confers partial LIF independence to ESC propagation^19^. In addition to Klf4, two other Klf family members, Klf2 and Klf5, are also specifically present in mESCs^20^. Triple knockdown of Klf4, Klf2 and Klf5 was shown to result in the impaired self-renewal of mESCs, whereas single knockdown of each gene did not lead to an apparent phenotype, suggesting a functional redundancy among Klf4, Klf2 and Klf5 (ref. 21).

As mentioned above, the pluripotent state of ESCs is intricately regulated by multiple signalling networks; however, the underlying mechanisms remain unclear. In this study, we apply a retroviral insertion vector pDisrup8-based screen for the identification of genes that are required for maintenance of mESC pluripotency. We identify membrane-associated ring finger (C3HC4) 5 (March5), a member of the MARCH family, as a previously undiscovered pluripotency maintaining factor. MARCH family proteins are characterized by a RING-CH domain and multiple trans-membrane domains. March5 has been recognized as an E3 ligase located at the mitochondria membrane, which is able to catalyse ubiquitination of the interacting proteins, such as Drp1, Mfn1/2 and mSOD1. It has been reported that March5 functions in the regulation of mitochondrial dynamics, reactive oxygen species (ROS) elimination and NF-kB signalling transduction^22^,^23^,^24^,^25^. Here, we show that March5 promotes mESC stemness via suppression of ERK activation. Our data thereby further broadens the landscape of regulatory networks in mESCs.

## Results

### March5 is involved in ES cell pluripotency maintenance

**Figure 1:**
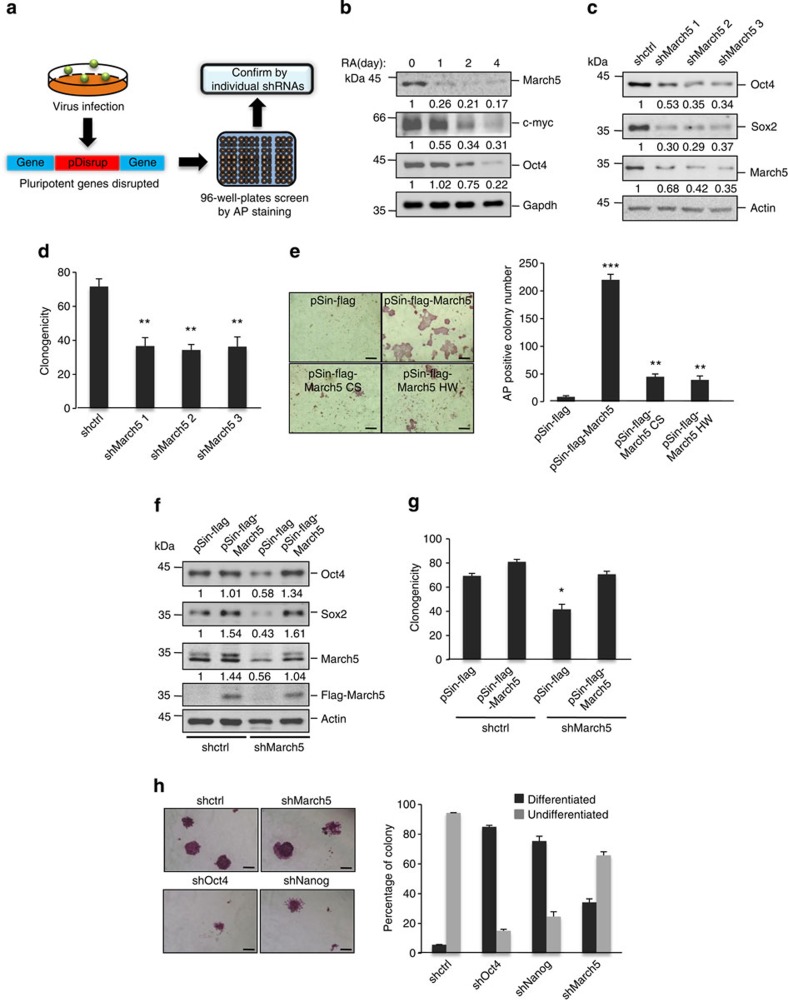
Identification of March5 as a pluripotency maintaining factor. (**a**) R1 cells infected with pDisrup8 retrovirus were selected with 10 μg ml^−1^ blasticidin for 48 h. ES cells with low AP activity were subjected to genomic DNA extraction, PCR amplification and DNA sequencing. The identified potential pluripotency maintaining genes are listed in Supplementary Table 1. (**b**) E14 cells were treated with RA. Cell lysates were analysed by Western blotting. The value of each band indicates the relative expression level after normalizing to the loading control GAPDH. (**c**) E14 cells were infected with lentiviruses expressing the indicated shRNAs. Seven days later, cell lysates were analysed by Western blotting. The value of each band indicates the relative expression level after normalizing to the loading control actin. (**d**) E14 cells were infected with lentiviruses expressing the indicated shRNAs. Five days later, ES cell colonies were stained for AP activity. AP-positive colonies were counted. Data are shown as the mean±s.d. from three independent experiments. **P<0.01. (**e**) E14 cells ectopically expressing the indicated proteins were cultured in N2B27 medium for four days before the colonies were stained for AP activity. The representative images from three independent experiments are shown. Scale bar represents 100 μm. The number of the AP-positive colonies is also shown as the mean±s.d. from three independent experiments. **P<0.01 and ***P<0.001. (**f**,**g**) E14 cells expressing March5 shRNA were infected with lentiviruses expressing shRNA-resistant Flag–March5. (**f**) Five days later, cell lysates were analysed by Western blotting. The value of each band indicates the relative expression level after normalizing to the loading control actin. (**g**) AP-positive colonies were counted. Data are shown as the mean±s.d. from three independent experiments. *P<0.05. (**h**) E14 cells were infected with lentiviruses expressing the indicated shRNAs. Four days later, ES cell colonies were stained for AP activity. The representative images from three independent experiments are shown. Scale bar represents 100 μm. The number of AP-positive colonies was counted and shown as the mean±s.d. from three independent experiments.

To define the signalling network that governs mESC identity, we carried out a phenotype-based screen using the pDisrup8 retrovirus. Previous studies have shown that the retroviral vector pDisrup8 contains a self-cleavage ribozyme and blasticidin^+^-encoding sequence. After insertion of pDisrup8 into the host genome, all of the transcripts upstream of the incorporated ribozyme-encoding sequence will be destroyed, and the downstream blasticidin^+^-coding sequence fused with endogenous messenger RNA (mRNA) allows expression of full-length blasticidin^+^ gene product (Supplementary Fig. 1a)^26^. After treatment with blasticidin for 48 h, cells carrying loss-of-function mutations will be alive. Therefore, pDisrup8 allows us to identify the genes that are potentially essential for ESC pluripotency maintenance. R1 mESCs were first infected with pDisrup8 retrovirus before they were treated with blasticidin. The live cells were then collected, re-plated into 96-well plates and stained for alkaline phosphatase (AP) activity after culturing in mESC medium for 7 days. The AP-negative clones were considered to be differentiated ES cells. These clones were individually collected, followed by genomic DNA extraction, PCR amplification and DNA sequencing to locate the retroviral insertion sites. By performing this systematic analysis (Fig. 1a), we identified 24 candidate genes (Supplementary Table 1), the inactivation of which may lead to mESC differentiation. We selected four genes (Supplementary Fig. 1b) for further validation; the other 20 genes are still in the process of validation. We found that knockdown of March5 led to decreased expression of stem cell pluripotency markers Nanog and Sox2 (Supplementary Fig. 1b), suggesting that March5 is important for stem cell pluripotency maintenance. We therefore focused on March5 for further study.

March5 has been associated with mitochondrial dynamics, ROS elimination and signalling transduction^22^,^23^,^24^,^25^. To examine the function of March5 in mESCs, we first compared the expression of March5 in mouse embryonic fibroblasts (MEFs) and R1 ES cells. March5 was highly expressed in R1 ES cells (Supplementary Fig. 1c). However, in response to retinoic acid (RA)-induced ES cell differentiation and during embryoid body formation, the expression of March5 was strongly suppressed (Fig. 1b and Supplementary Fig. 1d,e). These data imply that expression levels of March5 are correlated with the pluripotent state of ES cells. We next evaluated the effect of March5 on ES cell pluripotency. In agreement with results showing that knockdown of March5 decreased Oct4 and Sox2 expression (Fig. 1c and Supplementary Fig. 1f), March5 knockdown resulted in the differentiation of ES cells, as shown by decreased expression of pluripotency markers and increased expression of differentiation markers (Supplementary Fig. 1g). Similarly, knockdown of March5 strongly inhibited the clonogenicity of mESCs (Fig. 1d and Supplementary Fig. 1h). By contrast, ectopic expression of March5 markedly protected ES cells from differentiation, as shown by increased numbers of AP-positive colonies after March5 overexpression in both embryoid body formation and N2B27-induced differentiation conditions (Fig. 1e and Supplementary Fig. 1i). These data demonstrate an important function of March5 in the maintenance of ES cell pluripotency. Interestingly, E3 ligase-inactive March5 (H43W) and March5 (C65/68S)^23^,^27^, in which one conserved histidine or two conserved cysteine residues located in the Ring domain of March5 were mutated, failed to protect ES cells from N2B27-induced differentiation (Fig. 1e), indicating that March5 promotes ES cell pluripotency through its E3 ligase activity. Moreover, the function of March5 appears to rely on its mitochondrial localization, as a nuclear-localized March5-NLS fusion protein was unable to protect mESCs from differentiation (Supplementary Fig. 2).

To exclude the possibility of an off-target effect of March5 short hairpin RNA (shRNA), we generated a shRNA-resistant March5 expressing plasmid. As expected, exogenous expression of Flag–March5 almost completely restored pluripotency marker expression and clonogenicity in March5 knockdown E14 ES cells (Fig. 1f,g), indicating the specific effect of March5 on ES cell pluripotency. To further evaluate the importance of March5 in regulating ES cell pluripotency, the effects of March5, Oct4 and Nanog on ES cell pluripotency were compared. Knockdown of March5 consistently increased the percentage of differentiated ES cells, although to a lesser extent than knockdown of either Oct4 or Nanog (Fig. 1h). Together, these results demonstrate an important function of March5 in maintaining mouse ES cell pluripotency.

### March5 promotes somatic cell reprogramming

**Figure 2:**
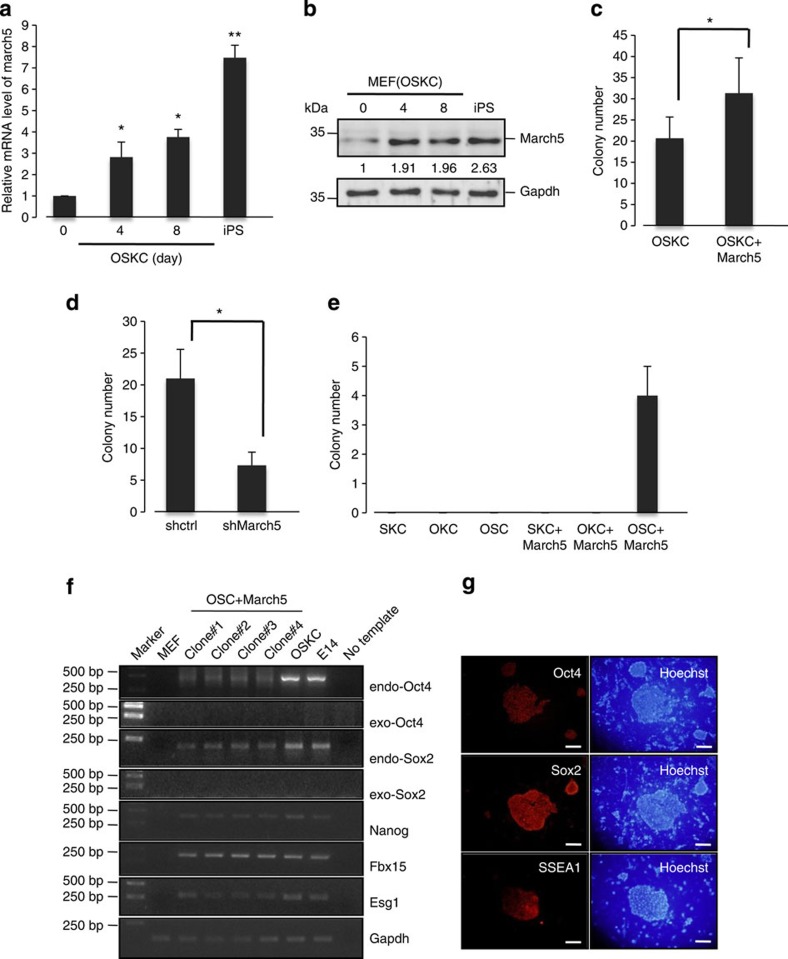
March5 increases somatic cell reprogramming efficiency. (**a**) Total RNA was extracted from MEF cells on day 0, 4 and 8 after transduction with OSCK (Oct4, Sox2, c-Myc and Klf4) and OSCK-derived iPS cells and analysed for March5 mRNA expression by real-time RT–PCR. Data are shown as the mean±s.d. from three independent experiments. *P<0.05 and **P<0.01. (**b**) Lysates from MEF cells on day 0, 4 and 8 after transduction with OSCK and OSCK-derived iPS cells were analysed by Western blotting to examine March5 protein expression. The value of each band indicates the relative expression level after normalizing to the loading control GAPDH. (**c**) MEF cells were introduced with OSCK together with lentiviruses expressing either March5 or control proteins. Fourteen days after transduction, the number of AP-positive colonies was counted. Data are shown as the mean±s.d. from three independent experiments. *P<0.05. (**d**) MEF cells were introduced with OSCK together with lentiviruses expressing either control or March5-specific shRNA. Fourteen days after transduction, the number of AP-positive colonies was counted. Data are shown as the mean±s.d. from three independent experiments. *P<0.05. (**e**) MEF cells were infected with lentiviruses expressing the indicated proteins. Fourteen days after infection, the number of AP-positive colonies was counted. Data are shown as the mean±s.d. from three independent experiments. (**f**) Total RNA from MEF cells, E14 cells, and the indicated colonies was subjected to semi-quantitative RT–PCR analysis for detection of expression of endogenous Oct4, Sox2, Nanog, Fbx15 and Esg1, and exogenous Oct4 and Sox2. Actin was used as an internal control. (**g**) Colonies after March5 and OSC transduction were stained for expression of Oct4, Sox2 and SSEA1. Scale bar represents 50 μm.

Given the important role of March5 in the regulation of ES cell pluripotency, we sought to determine whether March5 also regulates somatic cell reprogramming. We first examined March5 expression levels in MEF cells during reprogramming to induced pluripotent stem (iPS) cells. March5 was highly expressed in iPS cells but was expressed at low levels in MEF cells (Fig. 2a,b). Moreover, both mRNA and protein levels of March5 were increased during OSKC (Oct4, Sox2, Klf4 and c-Myc)-induced MEF cell reprogramming (Fig. 2a,b), indicating that March5 may facilitate reprogramming. To test this idea, we introduced March5 into MEF cells along with OSKC transduction. Ectopic expression of March5 indeed resulted in an increase in the AP-positive colonies (Fig. 2c). Conversely, knockdown of March5 markedly inhibited OSKC-induced reprogramming efficiency (Fig. 2d), implying that March5 is required for somatic cell reprogramming. Intriguingly, we found that Klf4, but not Oct4 and Sox2, can be replaced by March5 for induction of AP-positive colonies (Fig. 2e). Additionally, semi-quantitative reverse transcription (RT-PCR) analysis revealed that all of the examined OSCM (Oct4, Sox2, c-Myc and March5)-derived colonies expressed endogenous ES cell marker genes, including Oct4, Sox2, Nanog, Fbx15 and Esg1, whereas exogenous transgenes such as *Oct4* and *Sox2* were silenced (Fig. 2f). In addition, immunofluorescence analysis showed that these colonies were positive for ES cell markers, such as Oct4, Sox2 and SSEA1 (Fig. 2g). However, these cells were not able to form teratomas containing derivatives of three germ layers in nude mice (data not shown), indicating that induction of OSCM into MEF cells forms some reprogramming intermediates that are not fully pluripotent. Taken together, these findings strongly suggest that March5 promotes reprogramming of MEF cells, and also indicate March5 and Klf4 have at least partially overlapping functions in regulating somatic cell reprogramming.

### March5 is transcriptionally regulated by Klf4

**Figure 3:**
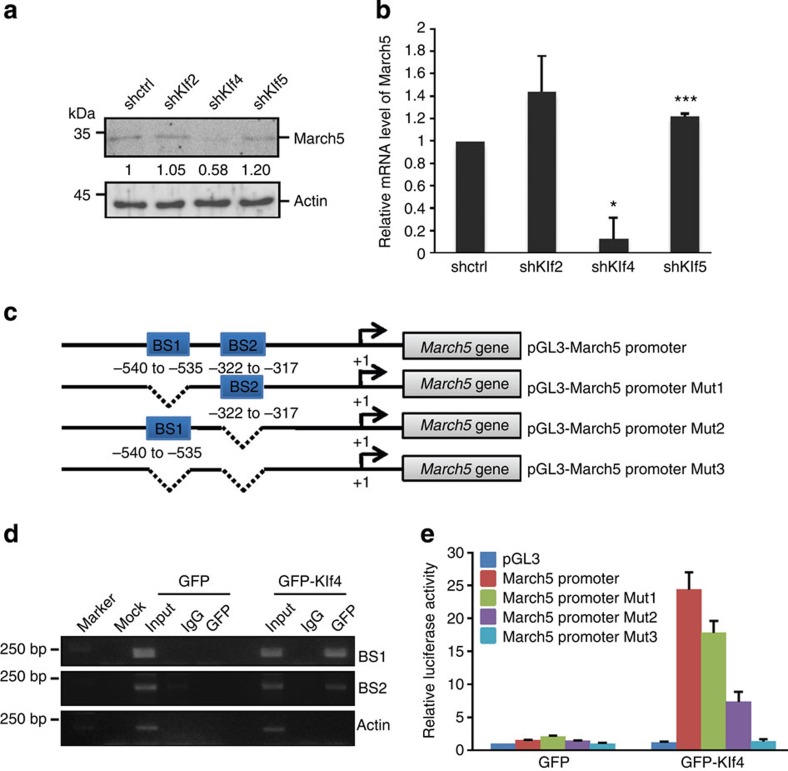
March5 is transcriptionally regulated by Klf4. (**a**) E14 cells were infected with lentiviruses expressing the indicated shRNAs. Four days after infection, cell lysates were analysed by Western blotting with anti-March5 antibody. The value of each band indicates the relative expression level after normalizing to the loading control actin. (**b**) E14 cells were infected with lentiviruses expressing the indicated shRNAs. Four days after infection, cell lysates were subjected to real-time RT–PCR analysis to examine March5 expression levels. Data are shown as the mean±s.d. from three independent experiments. *P<0.05 and ***P<0.001. (**c**) Schematic illustration of putative Klfs-binding sites in the *March5* gene promoter. pGL3-based wild type and multiple mutant reporter constructs used for luciferase assays are also shown. Mut1 and Mut2 indicate the promoter region (−750 to −1) of *March5* with deletion of BS1 and BS2, respectively. Mut3 indicates the promoter region (−750 to −1) of *March5* with both BS1 and BS2 deletion. (**d**) MEF cells were transfected with either GFP-Klf4 or GFP control vector. Twenty-four hours after transfection, cell lysates were subjected to ChIP analysis with anti-GFP antibody or an isotype-matched IgG. ChIP products were amplified by semi-quantitative PCR. Actin was used as a negative control. (**e**) HEK 293T cells were co-transfected with either GFP-Klf4 or GFP control vector together with the indicated reporter plasmids. Twenty-four hours later, reporter activity was measured and plotted after normalizing with respect to Renilla luciferase activity (mean±s.d.).

The finding that March5 can partially replace Klf4 to induce MEF cell reprogramming (Fig. 2e) led us to test the possibility that March5 may be regulated by Klf4. It has been shown that three members of Klf family proteins (Klf2, Klf4 and Klf5) are specifically present in mESCs^21^. To determine whether these three Klfs regulate March5 expression, we knocked down Klf2, Klf4 or Klf5 in E14 cells. The results showed that knockdown of Klf4, but not Klf2 or Klf5, greatly decreased both mRNA and protein levels of March5 (Fig. 3a,b and Supplementary Fig. 3a). Conversely, ectopic expression of Klf4 increased March5 expression (Supplementary Fig. 3b). These data indicate that March5 is specifically regulated by Klf4.

We next explored whether Klf4 could regulate March5 expression at the transcriptional level. We inspected the genomic sequence upstream of the gene coding for March5 by using the Genomatix suite of sequence analysis tools (MatInspector). Two putative Klfs-binding sites (BS1 and BS2) were found within the promoter region of the *March5* gene (Fig. 3c). The subsequent chromatin immunoprecipitation (ChIP) assays verified the association of Klf4, but not Klf2 or Klf5, with the chromatin fragments corresponding to BS1 and BS2 within the *March5* gene promoter (Fig. 3d and Supplementary Fig. 3c). In addition, we evaluated whether the Klfs-binding sites within the *March5* gene promoter confer Klf4-dependent transcriptional activity. DNA fragments containing wild type or the indicated mutant Klfs-binding sites were inserted into the promoter region of a firefly luciferase reporter plasmid (Fig. 3c). As expected, luciferase expression from the wild-type reporter was dramatically induced by Klf4, but not by Klf2 or Klf5 (Fig. 3e and Supplementary Fig. 3d). However, deletion of either BS1 or BS2 led to a decreased luciferase activity upon Klf4 induction (Fig. 3e). Additionally, luciferase activity from the mutant reporter containing both BS1 and BS2 deletion was not induced by Klf4 at all (Fig. 3e). These data therefore demonstrate that March5 is a direct transcriptional target of Klf4.

### March5 regulates ERK activation through Prka1a

**Figure 4:**
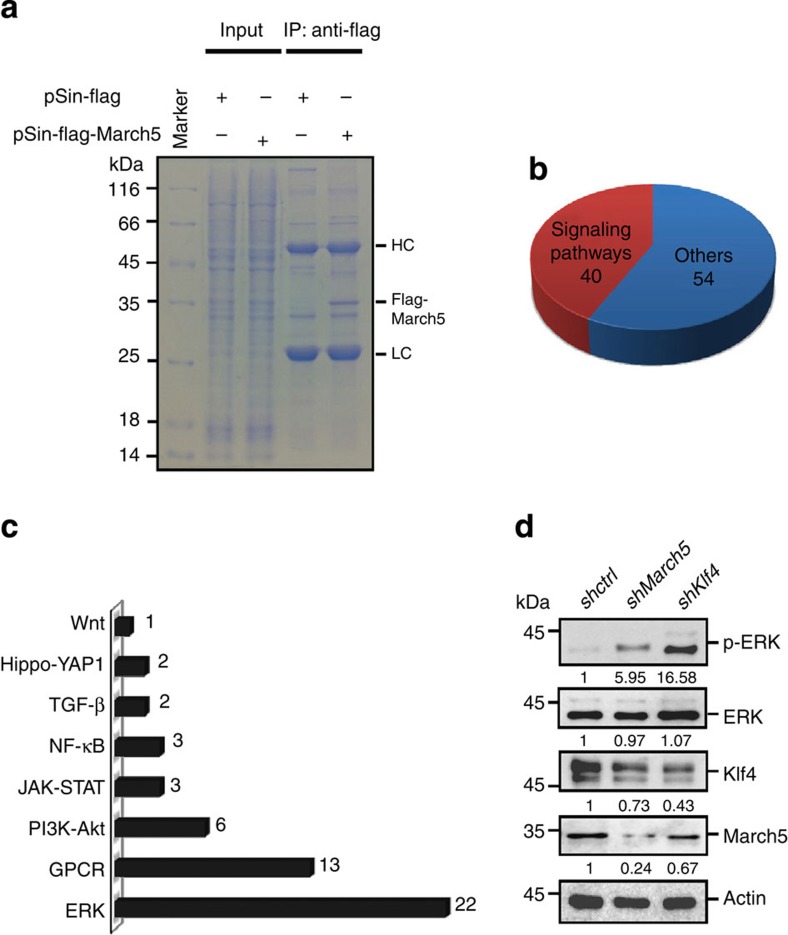
Identification of March5-interacting partners. (**a**) R1 ES cells were infected with lentiviruses expressing Flag–March5 or control proteins. Twenty-four hours after infection, cell lysates were immunoprecipitated with anti-Flag M2 affinity beads. After extensive washing, the bound proteins were eluted, resolved by SDS–PAGE, and visualized by Coomassie brilliant blue staining. The Flag–March5 band was indicated. (**b**,**c**) R1 ES cells were infected with lentiviruses expressing Flag–March5. Twenty-four hours after infection, cell lysates were immunoprecipitated with anti-Flag M2 affinity beads. After extensive washing, the bound proteins were eluted, and proteins associated with Flag–March5 were analysed by mass spectrometry. Ninety-four proteins were found to be specifically present in anti-Flag immunoprecipitates from Flag–March5-expressing R1 cells, but not from control R1 cells. The identified potential March5-interacting proteins were classified into two different categories (**b**). The number of candidate proteins involved in different signalling pathways is also shown (**c**). (**d**) E14 cells were infected with lentiviruses expressing the indicated shRNAs. Cells were maintained in ESCs culture medium in the presence of LIF as indicated for four days. Cell lysates were then analysed by Western blotting with the indicated antibodies. The value of each band indicates the relative expression level after normalizing to the loading control actin.

To further understand the mechanism by which March5 promotes stemness of ES cells, we sought to identify new March5-interacting partners using an affinity purification approach. Flag-tagged March5 or control proteins were expressed in R1 ES cells. Cell lysates were incubated with anti-Flag antibody, and the immunoprecipitated proteins were resolved by SDS–polyacrylamide gel electrophoresis (SDS–PAGE) (Fig. 4a). The subsequent mass spectrometry analysis revealed that 94 proteins were specifically present in anti-Flag immunoprecipitates from Flag–March5-expressing R1 cells, but not from control R1 cells (Supplementary Table 2). After performing bioinformatics analysis, we found that 40 of these 94 March5-interacting candidate proteins were previously reported to be involved in signalling transduction (Fig. 4b). We categorised 40 signalling transducers into eight different signalling pathways that have already been shown to regulate ES cell stemness (Fig. 4c and Supplementary Table 3). We unexpectedly found that 22 March5-interacting candidates fell into the category that contains ERK signalling pathway, implying that March5 could regulate the ERK signalling pathway. To test this possibility, we examined expression levels of phosphorylated ERK (Thr202/Tyr204) (p-ERK) in March5- and Klf4-knockdown E14 cells. Knockdown of either March5 or Klf4 indeed led to increased expression of p-ERK (Fig. 4d and Supplementary Fig. 4a).

**Figure 5:**
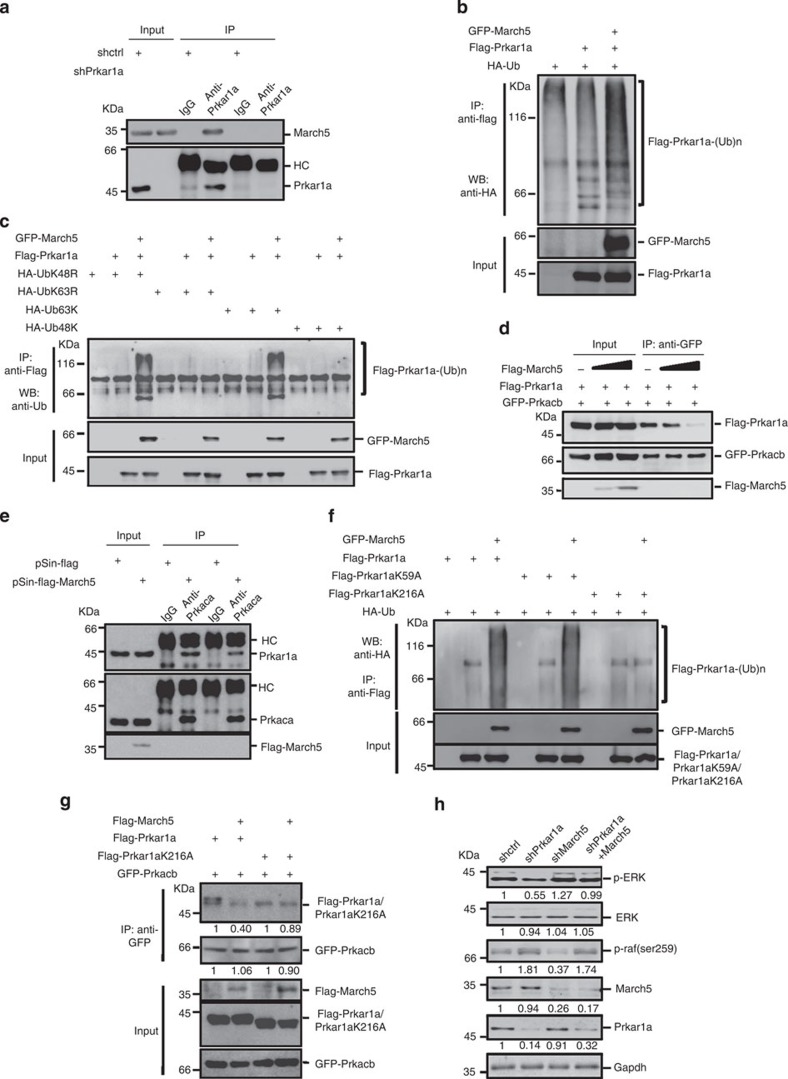
March5 catalyses polyubiquitination of Prkar1a. (**a**) Lysates from E14 cells infected with lentivirus expressing Prkar1a shRNA or control shRNA were subjected to co-immunoprecipitation analysis with anti-Prkar1a antibody or an isotype-matched IgG. The immunoprecipitates were analysed by Western blotting with the indicated antibodies. (**b**) HEK 293T cells were co-transfected with the indicated plasmids. Twenty-four hours after transfection, cells were treated with MG132 for an additional 6 h. Cell lysates were then subjected to immunoprecipitation, followed by Western blotting analysis with the indicated antibodies. (**c**) HEK 293T cells were co-transfected with the indicated plasmids. Twenty-four hours later, cells were treated with MG132 for an additional 6 h. Cell lysates were then subjected to immunoprecipitation, followed by Western blot analysis with the indicated antibodies. (**d**) HEK 293T cells were transfected with Flag-Prkar1a, GFP-Prkacb and increasing amounts of Flag–March5. Cell lysates were immunoprecipitated with anti-GFP antibody, followed by Western blot analysis. (**e**) E14 cells were infected with lentiviruses expressing Flag–March5 or control proteins. Cell lysates were immunoprecipitated with anti-Prkaca antibody or an isotype-matched IgG, followed by Western blot analysis. (**f**) HEK 293T cells were co-transfected with the indicated plasmids. Twenty-four hours later, cells were treated with MG132 for an additional 6 h. Cell lysates were then subjected to immunoprecipitation, followed by Western blot analysis with the indicated antibodies. (**g**) HEK 293T cells were transiently transfected with the indicated plasmids. Cell lysates were immunoprecipitated with anti-GFP antibody, followed by Western blot analysis. The value of each band indicates the relative expression level after normalizing to the input. (**h**) E14 cells were infected with lentiviruses expressing Prkar1a or March5-specific shRNA in the indicated combinations. Five days later, cell lysates were analysed by Western blotting with the indicated antibodies. The value of each band indicates the relative expression level after normalizing to the loading control actin.

We next determined how March5 regulates the ERK signalling pathway. Based on the mass spectrometry data, we tested whether March5 could interact with FGFR1 and ERK1, which are two classic members of ERK signalling transduction in mESCs. We found that neither FGFR1 nor ERK1 interacted with March5 (Supplementary Figs 4b and 4c). However, the co-immunoprecipitation assay verified an interaction between the endogenous March5 and Prkar1a (Fig. 5a). Prkar1a is a negative regulatory subunit of protein kinase A (PKA, a cAMP-dependent protein kinase A), and PKA has been shown to inhibit ERK activation through phosphorylation of Raf-1 at Ser259^28^. Therefore, our data suggest that March5 may regulate the ERK signalling pathway via interacting with Prka1a. Because it has been previously shown that March5 functions as an E3 ubiquitin ligase, we sought to determine whether March5 could be an ubiquitin E3 ligase for Prkar1a. By performing an *in vivo* ubiquitination assay, we showed that March5 indeed greatly enhanced Prkar1a ubiquitination (Fig. 5b). Polyubiquitination usually occurs at Lys^48^ and Lys^63^ of ubiquitin. To examine the type of Prkar1a polyubiquitination chain, we performed the assay with the ubiquitin mutants UbK48R and UbK63R, which eliminate Lys^48^- and Lys^63^-conjugated polyubiquitin chains, respectively. March5 was shown to greatly induce the polyubiquitination of Prkar1a in the presence of UbK48R but not UbK63R, suggesting that March5 promotes Lys^63^-linked polyubiquitination of Prkar1a (Fig. 5c). In support of this, March5 was able to induce polyubiquitination of Prkar1a in the presence of Ub63K but not Ub48K (Ub48K and Ub63K lack all lysine residues except K48 and K63, respectively) (Fig. 5c). K63-linked ubiquitination is not associated with proteasome-dependent degradation of target proteins. Instead, K63-mediated ubiquitination has been demonstrated to alter the function of the modified proteins, particularly influencing signalling complex formation^29^,^30^. We therefore examined whether March5-mediated k63-linked polyubiquitination of Prkar1a could affect the association of Prkar1a with PKA catalytic subunit Prkaca or Prkacb. Our data showed that with increasing expression of March5, the interaction of Prka1a with PKA catalytic subunit was concurrently decreased (Fig. 5d,e), indicating that March5 facilitates the disassociation of Prkar1a from the PKA catalytic subunit via Prkar1a polyubiquitination. To determine how March5-mediated ubiquitination of Prkar1a affects the interaction with PKA catalytic subunit, we sought to identify the ubiquitination site(s) in Prkar1a. By using an bioinformatics analysis^31^, we found K59 and K216 of Prkar1a as potential ubiquitination sites. We therefore performed an *in vivo* ubiquitination assay with Prkar1a K59A or Prkar1a K216A mutant proteins. The results showed that March5 greatly promoted ubiquitination of both Prkar1a and Prkar1a K59A, but did not show similar effect on Prkar1a K216A (Fig. 5f), suggesting that March5-mediated Prkar1a ubiquitination occurs at K216. Importantly, we showed that the interaction of Prkar1a K216A with Prkacb was not affected by ectopic expression of March5 (Fig. 5g). These results indicate that March5 promotes ubiquitination of Prkar1a at K216, thus leading to the dissociation of Prkar1a from PKA catalytic subunit.

To further determine whether March5 affects ERK activation through Prkar1a, we knocked down both March5 and Prkar1a in E14 cells. Knockdown of March5 consistently led to increased expression of p-ERK, which was accompanied by decreased levels of phosphorylated Raf (Fig. 5h). However, these March5 knockdown effects were partially reversed by the simultaneous knockdown of Prkar1a (Fig. 5h), indicating that Prkar1a mediates the effect of March5 on ERK activation. Collectively, these data suggest that March5 interacts with Prkar1a and promotes the non-classical Lys63-linked ubiquitination of Prkar1a. In turn, this relieves the inhibitory effect of Prkar1a on PKA and enhances PKA activity, thereby leading to the inhibition of ERK.

### March5 regulates pluripotency via the Prkar1a-ERK pathway

**Figure 6:**
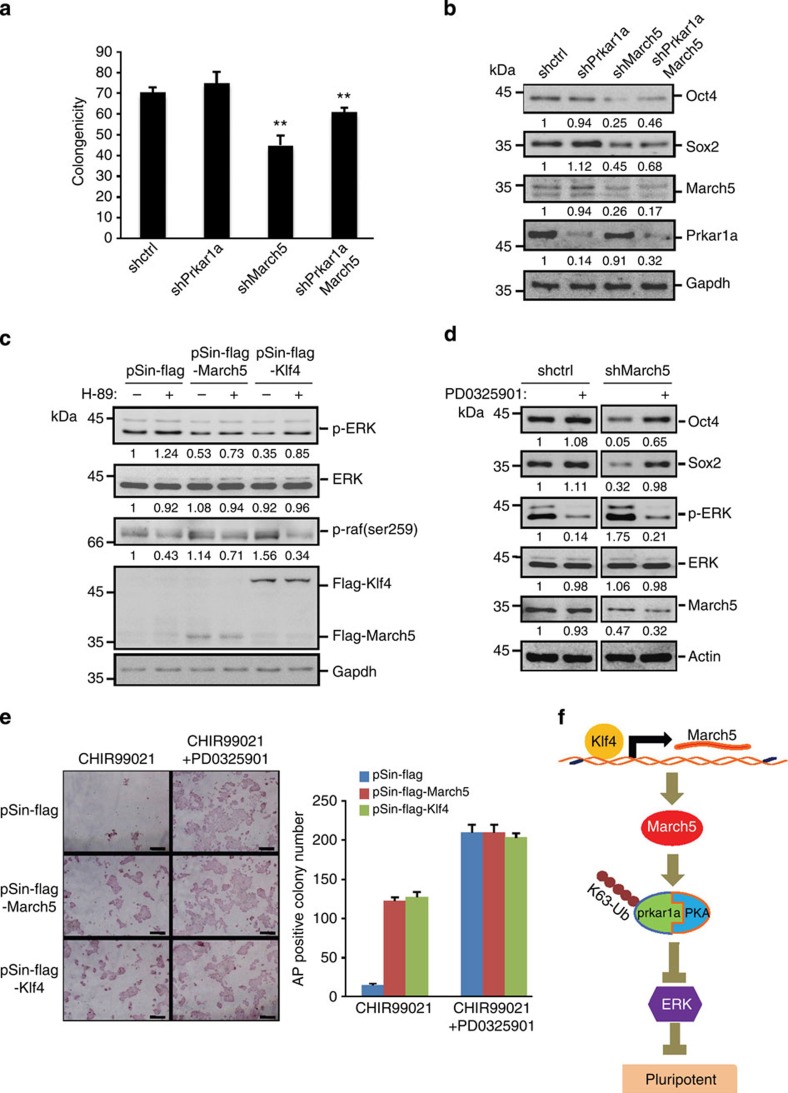
ES cell pluripotency is regulated by the Klf4–March5–ERK pathway. (**a**,**b**) The differentiation phenotype of March5 knockdown ES cells was rescued by Prkar1a knockdown. E14 cells were infected with lentiviruses expressing Prkar1a or March5-specific shRNA in the indicated combinations. (**a**) Five days later, the number of AP-positive colonies was counted and is shown as the mean±s.d. from three independent experiments. **P<0.01. (**b**) Cell lysates were also analysed by Western blotting with the indicated antibodies. The value of each band indicates the relative expression level after normalizing to the loading control GAPDH. (**c**) E14 cells-expressing Flag–March5, Flag–Klf4 or control proteins were treated with PKA inhibitor H-89 for 24 h. Cell lysates were analysed by Western blotting. The value of each band indicates the relative expression level after normalizing to the loading control GAPDH. (**d**) E14 cells-expressing control or March5-specific shRNA were treated with the MEK inhibitor PD0325901 for four days. Cell lysates were subjected to Western blot analysis with the indicated antibodies. The value of each band indicates the relative expression level after normalizing to the loading control actin. (**e**) E14 cells expressing Flag–March5, Flag–Klf4 or control proteins were plated on matrigel-coated dishes at clonal density in mESC culture medium for one day. The culture medium was then changed to N2B27 medium supplemented with either GSK3β inhibitor CHIR99021 alone or GSK3β inhibitor CHIR99021 and MEK/ERK inhibitor PD0325901. After three passages, the ES cell colonies were stained for alkaline phosphatase activity. The shown images are representative from three independent experiments. The number of AP-positive colonies was also counted and shown as the mean±s.d. from three independent experiments. (**f**) A proposed model illustrating the role of the Klf4–March5–ERK pathway in maintaining mESC pluripotency.

Given that March5 inhibits activation of ERK through Prkar1a, we sought to determine whether March5 could regulate ES cell pluripotency via the Prkar1a–ERK pathway. We showed that knockdown of March5 suppressed the clonogenecity of E14 cells and decreased expression levels of Oct4 and Sox2 (Fig. 6a,b). However, knockdown of Prkar1a greatly restored the clonogenecity and expression levels of Oct4 and Sox2 in March5 knockdown E14 cells (Fig. 6a,b), indicating that March5 promotes ES cell pluripotency through Prkar1a. Because Prkar1a is a negative regulator of PKA, we therefore examined whether Prkar1a-mediated PKA activation is essential for the effect of March5 on ES cell pluripotency. We treated E14 cells with H-89, a specific inhibitor of PKA. H-89 treatment resulted in the differentiation of E14 cells, which was correlated with decreased levels of Oct4 and Sox2, and increased levels of p-ERK in these cells (Supplementary Fig. 5a,b) Treatment with H-89 also restored p-ERK expression in March5- or Klf4-overexpressing E14 cells (Fig. 6c). Moreover, blocking MEK/ERK activity by the inhibitor PD0325901 greatly reversed March5 knockdown-mediated decrease in expression of Oct4 and Sox2 (Fig. 6d). These data suggest that the Prkar1a–ERK pathway mediates the effect of March5 on stem cell pluripotency.

It has been reported that mESCs can be maintained in N2B27 differentiation medium in the presence of MEK/ERK inhibitor PD0325901 and GSK3 inhibitor CHIR9902121 (2i)^32^, suggesting the inhibitory roles of MEK/ERK and GSK3 signaling in maintaining mESC pluripotency. This 2i system provided an ideal model to further evaluate the importance of the March5-Prkar1a-ERK pathway in regulating ES cell pluripotency. Consistent with previous reports^32^,^33^,^34^, E14 and R1 ES cells were able to self-renew in N2B27 medium supplemented with the GSK3 inhibitor CHIR99021 for three passages, although they underwent progressive differentiation (Fig. 6e and Supplementary Fig. 6). Intriguingly, ectopic expression of March5 or Klf4 greatly increased the number of ES cell colonies in CHIR99021-containing N2B27 medium, but did not show any significant additive effect in N2B27 medium supplemented with both CHIR99021 and PD0325901 (Fig. 6e and Supplementary Fig. 6), suggesting that March5 is able to replace MEK/ERK inhibitor to maintain mESC identity in N2B27 medium. Taken together, these results indicate that March5 regulates mESC pluripotency via the Klf4–March5–Prkar1a/PKA–ERK pathway.

## Discussion

In this study, we identify March5 as a novel mESC pluripotency maintaining factor. We demonstrate that March5 is able to bind and ubiquitinate Prkar1a via a non-canonical ubiquitination through K63-linked ubiquitin chains. Ubiquitination of Prkar1a promotes disassociates from the PKA catalytic subunit, thereafter leading to activation of PKA. It has been shown that in cancer cells, PKA indirectly inhibits MEK/ERK activation through phosphorylation of Raf-1 at serine 259 (ref. 28). We show that the PKA-mediated MEK/ERK inhibition also occurs in mESCs. ERK has been shown to be one of the most important pathways in mESCs^32^,^35^. FGF4-triggered ERK activation leads to the loss of naive pluripotency of mESCs and hypoblast specification of the inner cell mass^36^,^37^. By contrast, inhibition of the ERK pathway can prevent mESCs from differentiating and replace LIF for mESC culture in combination with a GSK3 inhibitor^32^,^34^,^38^. Therefore, it is not surprising that March5 exerts its function in promoting mESC stemness by inhibiting ERK activation. It is important to mention that by using an affinity purification approach, we have identified 22 March5-interacting candidates that are involved in the regulation of the ERK signalling pathway. It will be interesting to further determine if March5 could inhibit ERK activation through other binding partners in addition to Prkar1a, thereby contributing to the maintenance of mESC pluripotency.

It has been previously reported that a MEK/ERK inhibitor alone is incapable of supporting clonal colony formation of mESCs, whereas GSK3 inhibitor alone does support the formation of partially undifferentiated colonies although it cannot sustain ES cell self-renewal in long-term culture^32^,^33^,^34^. In agreement with these findings, we showed that ES cells cultured with MEK/ERK inhibitor PD0325901-containing N2B27 medium died after first passage. However, ES cells were able to self-renew in N2B27 medium supplemented with GSK3 inhibitor CHIR99021 for up to three passages. By using this stringent assay, March5 was shown to function as a replacement for the MEK/ERK inhibitor in maintaining pluripotency of mESCs. Our data suggest an intriguing possibility that March5 could confer long-term self-renewal of mESCs in combination with a GSK3 inhibitor.

As mentioned above, March5 is an ubiquitin E3 ligase located in the mitochondria with four membrane-spanning segments. One interesting question is whether the E3 ligase activity and the mitochondrial localization are required for March5 function. Here, we show that the function of March5 in stemness of mESCs relies on its E3 ligase activity and mitochondrial localization. An additional question is whether March5 could regulate pluripotency through other signaling pathways in addition to inhibiting ERK activation. March5 is a multifunctional protein and has been implicated in the regulation of mitochondrial dynamics, ROS elimination and NF-kB signalling transduction^22^,^23^,^24^,^25^. It has been shown that ROS enhances ESC differentiation^39^,^40^. Consistent with this, RA treatment led to the increased levels of ROS in E14 cells (Supplementary Fig. 7a). We therefore sought to investigate whether March5 could regulate pluripotency by eliminating ROS. We showed that March5 knockdown increased ROS levels of E14 cells (Supplementary Fig. 7b). March5 knockdown-enhanced ROS production led to the impaired pluripotency in E14 cells, which was rescued by treatment of *N*-acetyl cysteine, a ROS scavenger (Supplementary Fig. 7d). These findings suggest that March5 may regulate mESC pluripotency by reducing ROS production. It has also been reported that knockdown of growth factor erv1-like (Gfer) induces mESC differentiation by increasing expression levels of mitochondrial fission protein Drp1 (ref. 41). Given that March5 is an ubiquitin E3 ligase for Drp1, it is possible that March5 may also promote pluripotency of mESCs through degrading Drp1 and regulating mitochondrial dynamics.

Klf4 is one of the well-known Yamanaka factors. Here, we show that March5 is a direct transcriptional target of Klf4. It has been recognized that there are three members of Klf family proteins (Klf2, Klf4 and Klf5) expressed in mESCs^21^. However, our data show that expression levels of March5 are only regulated by Klf4, and not Klf2 and Klf5, suggesting the specificity of Klf4 in the regulation of March5 expression. Functionally, March5 is able to partially replace Klf4 for somatic cell reprogramming, yet the efficiency induced by March5 is lower than that induced by Klf4, implying that Klf4 may promote somatic cell reprogramming through other factors in addition to March5. In support of this idea, we found that although OSCM-reprogrammed cells successfully express ES cell marker genes (Fig. 2f,g), these cells cannot form three germ layers-containing teratomas, indicating that March5 has only the ability to initiate the reprogramming process, but cannot fully substitute Klf4 in this context. It is of interest to note that while knockdown of March5 leads to mESC differentiation, Klf4-deficient ES cells merely show mild changes in their stem cell identity^21^. This inconsistency implies that March5 may be regulated by other pluripotent factors.

LIF is a potent factor that supports ESC proliferation by activation of transcription factor STAT3^42^. In response to active LIF-STAT3 signalling, Klf4 is strongly induced^19^. Induced expression of Klf4 has been shown to confer partial LIF independence to ESC propagation^19^. In our study, ectopic expression of Klf4 is able to replace a MEK/ERK inhibitor and maintain mESC identity in the absence of LIF. Interestingly, this effect of Klf4 can be recapitulated by March5 overexpression. Given that March5 is a direct target of Klf4, it is conceivable that the Klf4–March5 axis has an important role in mediating the effect of LIF on mESC pluripotency maintenance.

On the basis of our data, we propose a model depicting an essential function of March5 in the regulation of mESC identity (Fig. 6f). As a transcriptional target of Klf4, March5 interacts and ubiquitinates Prkar1a, which relieves the inhibitory effect of Prkar1a on PKA and activates PKA, thereafter leading to the inhibition of ERK activity and promoting stemness of mESCs.

## Methods

### Reagents and antibodies

The following reagents used in this study were purchased from the indicated sources: CHIR99021 (Millipore, 3 μM), PD0325901 (Sigma, 1 μM), H-89 (Beyotime, 10 μM), antibodies against GAPDH (Santa Cruz, 1:1,000), Actin (Cell Signaling, 1:1,000), green fluorescent protein (GFP; MBL 1:4,000), Flag (Sigma 1:4,000), march5 (Abcam, 1:500), Prkar1a (Cell Signaling, 1:1,000), ERK (Cell Signaling, 1:1,000), p-ERK (Thr202/Tyr204) (Cell Signaling, 1:1,000), phosphorylated Raf (Ser259) (Santa Cruz, 1:1,000), Oct4 (Santa Cruz, 1:1,000) and Sox2 (Millipore, 1:1,000), SSEA1 (Santa Cruz, 1:50), HRP-conjugated secondary antibodies against mouse and rabbit IgG (Promega). Ubiquitin, E1, UbcH5a and 1 × energy regeneration solution were purchased from Boston Biochem (USA).

### Cell culture

Mouse embryonic stem cells (ESCs) were maintained in DMEM supplemented with 10% foetal bovine serum (FBS), 10% Knockout Serum Replacement, 2 mM L-glutamine, 100 μM non-essential amino acids, 0.1 mM β-mercaptoethanol, 1 mM sodium pyruvate (Invitrogen) and 1,000 U ml^−1^ LIF (Millipore) on gelatin-coated plates. For serum-free culture, we used N2B27 medium (Invitrogen) containing 2i inhibitors at the indicated concentration. MEF and HEK293T cells were cultured in DMEM supplemented with 10% FBS and 100 μg ml^−1^ streptomycin (Invitrogen).

### *In vitro* differentiation of mouse ESCs

To induce mouse ESCs differentiation *in vitro*, mouse ESCs grown on 0.1% gelatin-coated plate, maintained in ESC culture media lacking LIF and treated with 10^−7^ M RA (Sigma). Embryoid body formation was induced by plating mouse ESCs into non-adherent conditions in ES media lacking LIF.

### AP staining

Mouse ESCs were fixed in 4% paraformaldehyde at room temperature. After rinsing two times with PBS, ESCs were stained using the Alkaline Phosphatase Detection Kit (Millipore) according to the manufacturer's instruction.

### Colony-forming assay

Colony-forming assay was performed as previously described^16^. Briefly, ES cells were trypsinized to obtain a single cell suspension and 600 cells were plated per 10 cm^2^ well. After 6 days, colonies were stained for AP activities and divided into two categories: differentiated and undifferentiated.

### Induction of pluripotent stem cells

MEF cells were isolated from E13.5 embryos and induction of pluripotent stem cells was carried out as previously described^43^. Briefly, plat-E packaging cells were transfected with pMXs plasmids-expressing mouse Oct4, Sox2, Klf4 and c-Myc. Virus-containing culture medium were collected 36- and 60-h post transfection, and two rounds of infection were performed. Three or four days after virus infection, MEF cells were harvested and plated on mitomycin-C-treated MEF feeder cells, and the culture medium was changed to mouse embryonic stem cell medium. AP-positive clones were counted 14 days after virus infection.

### Plasmid construction

Primers used to generate plasmids encoding March5, Klf4 and Prkar1a were listed in Supplementary Table 4. The cDNAs were amplified by RT–PCR using total RNA from R1 ES cells.

### Dual-luciferase reporter assay

HEK293T cells were transiently transfected with the indicated plasmids using Lipofectamine 2000 (Invitrogen). The 750-bp DNA fragment containing the potential Klfs-binding sequence was amplified by PCR. The PCR product was then cloned into pGL3-Basic vector (Promega). The luciferase reporter assay was performed according to the manufacturer's instruction (Promega). The relative luciferase activities were calculated by normalizing the firefly luciferase activity to *Renilla* luciferase activity. The represented data are the mean±s.d. from at least three independent experiments.

### Real-time RT–PCR

Real-time RT–PCR was performed as previously described^44^. Briefly, total RNA was isolated using Trizol (Ambion). One microgram of total RNA was used to synthesize cDNA using a PrimeScriptTM RT reagent kit (Takara, DRR037A) according to the manufacturer's instruction. Real-time PCR was performed using SYBR premix EX Taq (TaKaRa) and ROX Reference Dye (ROX), and analysed with Stratagene Mx3000p (Agilent Technologies). The primers used for real-time RT–PCR were listed in Supplementary Table 4.

### Western blot analysis and co-immunoprecipitation

Western blot analysis was performed as previously described^44^. Briefly, cells were harvested, boiled in 1 × SDS loading buffer and resolved on SDS–PAGE. For co-immunoprecipitation, cells were lysed in IP lysis buffer (0.5% NP-40, 150 mM NaCl, 20 mM HEPES, pH 7.5, 2 mM EDTA and 1.5 mM MgCl_2_) supplemented with protease inhibitor cocktail for 1 h on ice. Cell lysates were incubated with protein A/G-sepharose beads coated with the indicated antibodies at 4 °C for 4 h. The immunoprecipitates were then subjected to Western blot analysis. Scans of uncropped blots are shown in Supplementary Figs 8 and 9.

### Immunofluorescence

iPS cells were seeded on 0.1% gelatin-coated plate. Four days after seeding, cells were fixed with 4% paraformaldehyde, permeabilized with 0.1% Triton-X-100 in PBS, and blocked with 1% BSA in PBS. Cells were then incubated at room temperature with anti-Oct4 (5 μg ml^−1^), anti-Sox2 (10 μg ml^−1^) or anti-SSEA1 (5 μg ml^−1^) antibody for 2 h and with rhodamine-conjugated anti-mouse or rabbit IgG secondary antibody for an additional 1 h. The cells were washed twice with PBS containing 0.1% Tween 20 and stained with Hoechst 33342 (Sigma). Images were captured with an Olympus fluorescence microscope.

### RNA interference

To generate lentiviruses expressing shRNAs, HEK 293 T cells grown on a 6-cm dish were transfected with 2 μg of shRNA (cloned in PLKO.1) or control vector, 2 μg of pREV, 2 μg of pGag/Pol/PRE and 1 μg of pVSVG. Twelve hours after transfection, cells were cultured with DMEM medium containing 20% FBS for an additional 24 h. The culture medium containing lentivirus particles was filtered through a 0.45 μm polyvinylidene difluoride filter (Millipore) and incubated with mouse ESCs supplemented with 4 μg ml^−1^ polybrene (Sigma) for 12 h, followed by selection with 5 μg ml^−1^ puromycin for another 24 h. The knockdown efficiency was evaluated by Western blot analysis and real-time RT–PCR analysis. The shRNA sequences are listed in Supplementary Table 4.

### Protein overexpression in mouse ESCs

To generate lentiviruses expressing the indicated proteins, HEK293T cells grown on a 6-cm dish were transfected with 2 μg of pSin-based construct, 1 μg of pmd2.g and 2 μg of pspax2. Twelve hours after transfection, cells were cultured with DMEM medium containing 20% FBS for an additional 24 h. The culture medium containing lentivirus particles was filtered through a 0.45 μm polyvinylidene difluoride filter and incubated with mouse ESCs in the ESC culture medium supplemented with 4 μg ml^−1^ polybrene for 12 h, followed by selection with 5 μg ml^−1^ puromycin for another 24 h.

### Identification of March5-interacting proteins

R1 ES cells were infected with lentiviruses expressing Flag–March5. Twenty-four hours after infection, cells were lysed in IP lysis buffer (0.5% NP-40, 150 mM NaCl, 20 mM HEPES, pH 7.5, 2 mM EDTA and 1.5 mM MgCl_2_) supplemented with protease inhibitor cocktail for 1 h on ice. Cell lysates were immunoprecipitated with anti-Flag M2 affinity beads. After extensive washing, the bound proteins were eluted with 3 × Flag peptide, and proteins associated with Flag–March5 were analysed by mass spectrometry.

### *In vivo* ubiquitination assay

HEK293T cells were transfected with the indicated plasmids. Twenty-four hours after transfection, cells were treated with 20 μM MG132 for an additional 6 h. *In vivo* ubiquitination assay was then performed according to the procedure described previously^45^. Briefly, cells were lysed in ubiquitination assay buffer (0.5% NP-40, 0.1% SDS, 150 mM NaCl, 20 mM HEPES, pH 7.5, 2 mM EDTA, 1.5 mM MgCl_2_ and 20 μM MG132) supplemented with protease inhibitor cocktail for 1 h on ice. Cell lysates were incubated with anti-Flag M2 affinity beads at 4 °C for 4 h. The immunoprecipitates were then subjected to Western blot analysis to examine Prkar1a ubiquitination.

### ChIP assay

ChIP assay was carried out essentially as described previously^46^. Briefly, MEF cells individually expressing GFP, GFP-Klf2, GFP-Klf4 or GFP-Klf5 were first cross-linked with 1% formaldehyde for 10 min at room temperature. The reaction was stopped by adding 0.125 M glycine. Nuclei were then isolated and re-suspended in lysis buffer (50 mM Tris-HCl (pH 8.1), 10 mM EDTA, 1% SDS and protease inhibitors). Cell lysates were incubated with protein A/G-sepharose beads conjugated with either anti-GFP antibody or control IgG. The bound DNA fragments were eluted and amplified by PCR.

### ROS measurement

Mitochondrial ROS production was measured by staining cells with 1 mM dichlorodihydrofluorescein diacetate (Beyotime) at 37 °C for 30 min in DMEM. Stained cells were filtered and analysed immediately in a FACScan flow cytometer (BD Bioscience).

### Reproducibility

All the data were repeated at least three times. The Western blot and ChIP analyses were representatives of three independent experiments.

### Statistical analysis

Statistical analysis was carried out using Microsoft Excel software and GraphPad Prism to assess differences between experimental groups. Statistical significance was analysed by Student's *t*-test and expressed as a *P*-value. *P*-values <0.05 were considered to be statistical significant.

## Additional information

**How to cite this article**: Gu, H. *et al*. Mitochondrial E3 ligase March5 maintains stemness of mouse ES cells via suppression of ERK signalling. *Nat. Commun.* 6:7112 doi: 10.1038/ncomms8112 (2015).

## Supplementary Material

Supplementary Information Supplementary Figures 1-9 and Supplementary Tables 1-4
