# Supplementary material for: Mitochondrial E3 ligase March5 maintains stemness of mouse ES cells via suppression of ERK signalling
Source: Nat Commun. 2015 Jun 2;6:7112. doi: 10.1038/ncomms8112 (PMC4458872; doi:10.1038/ncomms8112)
Supplement: Supplementary Information — Supplementary Figures 1-9 and Supplementary Tables 1-4 [file ncomms8112-s1.pdf]

Supplementary Figure 1

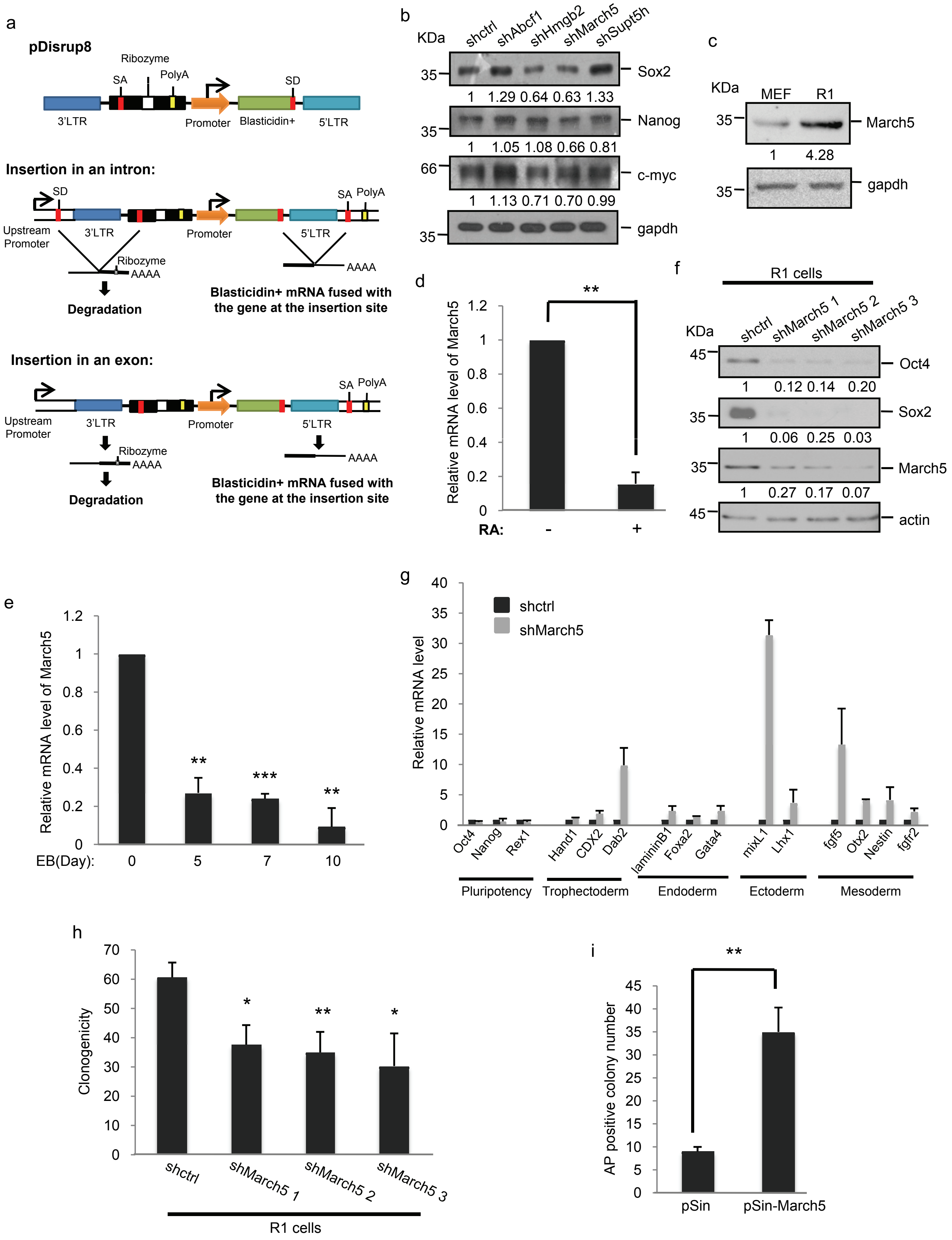

Supplementary Figure 1

(a) Schematic illustration of pDisrup8 retroviral vector-mediated insertional mutagenesis. The retroviral vector pDisrup8 contains a self-cleavage ribozyme sequence. Any transcript containing this ribozyme sequence will be degraded. Expression of blasticidin gene is dependent on the downstream poly(A) sequence from the endogenous gene. Therefore, blasticidin resistance can be applied for selection of the cells with a viral insertion in either an intron or exon of gene. If the viral insertion occurs in an intron of endogenous gene, the upstream gene SD will spice with the vector SA and the transcript will stop at the vector poly(A). The ribozyme located 3' to the SA sequence will then cleave the transcript and no gene product will be produced. The vector SD will splice with endogenous SA to generate mRNA capable of expressing blsticidin gene product. If the viral insertion occurs in an exon of endogenous gene, the transcript initiated by the gene will end at the vector poly(A). The ribozyme will destroy this transcript and no gene product will be produced. However, the blasticidin gene product will be produced normally. SA represents splicing acceptor, SD represents splicing donor.

(b) R1 cells were infected with lentiviruses expressing the indicated shRNAs. Five days after infection, cell lysates were subjected to Western blot analysis with the indicated antibodies. The value of each band indicates the relative expression level after normalizing to the loading control actin.

(c) Protein levels in MEF and R1 ES cells were examined by Western blot analysis with anti-March5 antibody. The value of each band indicates the relative expression level after normalizing to the loading control gapdh.

(d) E14 cells were treated with or without 10^{-7}M RA for 4 days. Total RNA was then exacted from these cells and subjected to real-time RT-PCR analysis to examine March5 mRNA levels. Data are shown as mean±SD from three independent experiments. ** indicates $P < 0.01$.

(e) E14 cells were plated into non-adherent conditions for the indicated periods of time to induce embryoid body (EB) formation. Total RNA was then analyzed by real-time RT-PCR to determine March5 mRNA expression. Data are shown as mean±SD from three independent experiments. ** and *** indicates $P < 0.01$ and $P < 0.001$, respectively.

(f) R1 cells were infected with lentivirues expressing control or three different sets of March5 specific shRNAs. Seven days later, cell lysates were subjected to Western blot analysis with the indicated antibodies. The value of each band indicates the relative expression level after normalizing to the loading control actin.

(g) Lineage marker expression after knocking down March5 in E14 cells. E14 cells were infected with lentiviruses expressing either control or March5 specific shRNA. Seven days after infection, cell lysates were analyzed by real-time RT-PCR. Data are shown as mean±SD from three independent experiments.

(h) R1 cells were infected with lentiviruses expressing control or the indicated March5 shRNAs. Five days after infection, AP-positive colonies were counted. Data are shown as mean±SD from three independent experiments. * and ** indicates $P < 0.05$ and $P < 0.01$, respectively.

(i) E14 cells exogenously expressing March5 or control proteins were cultured in non-adherent conditions for 12 days to induce embryoid body (EB) formation. The EBs were then dissociated and cultured in ES cell culture medium. Five days later, numbers of ESC-like colonies were analyzed by AP staining. Data are shown as mean±SD from three independent experiments. ** indicates $P < 0.01$.

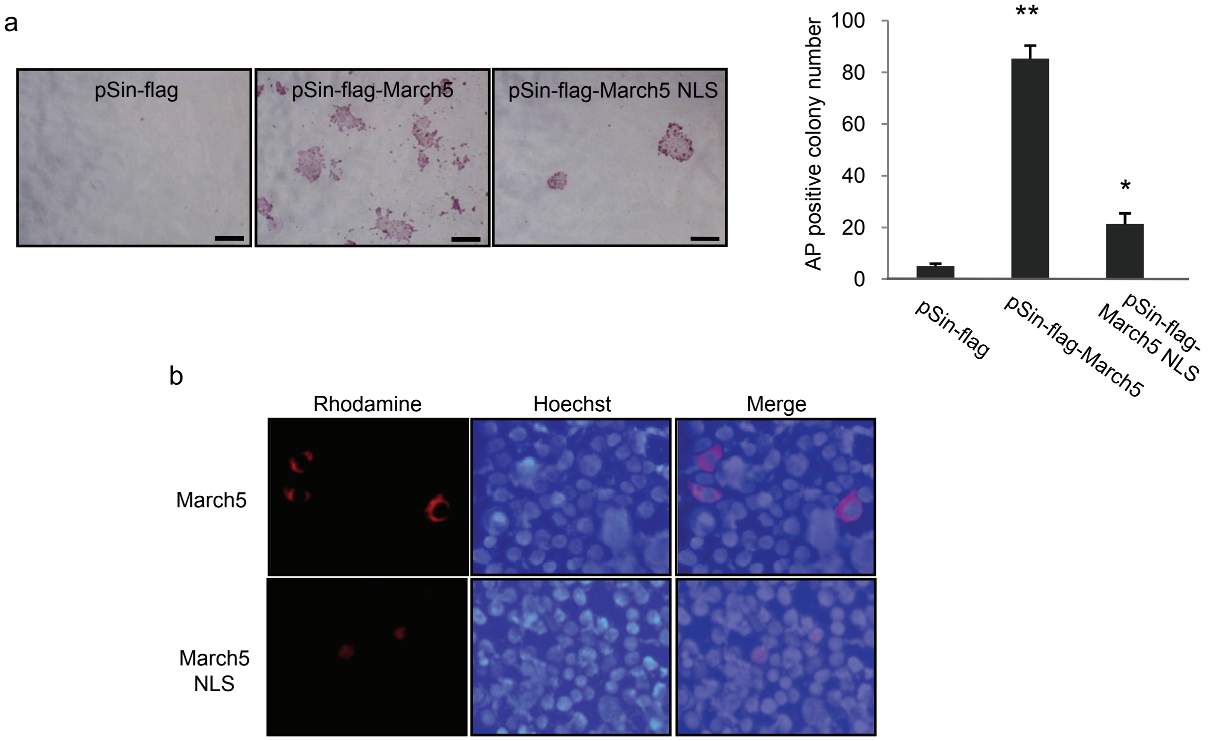

(a) E14 cells ectopically expressing Flag-March5, March5NLS or control proteins were cultured in N2B27 medium for 4 days before the colonies were stained for alkaline phophatase activity. The representative images from three independent experiments are shown. Scale bar represents 100μm. The number of the AP-positive colonies is also shown as mean±SD of three independent experiments. * and ** indicates P < 0.05 and P < 0.01, respectively.

(b) To confirm March5-NLS fusion protein is able to successfully localize in nucleus, HEK 293T cells were tansfected with either Flag-March5 or Flag-March5NLS, followed by immunofluorescence analysis to examine the cellular localization of March5 and March5NLS proteins.

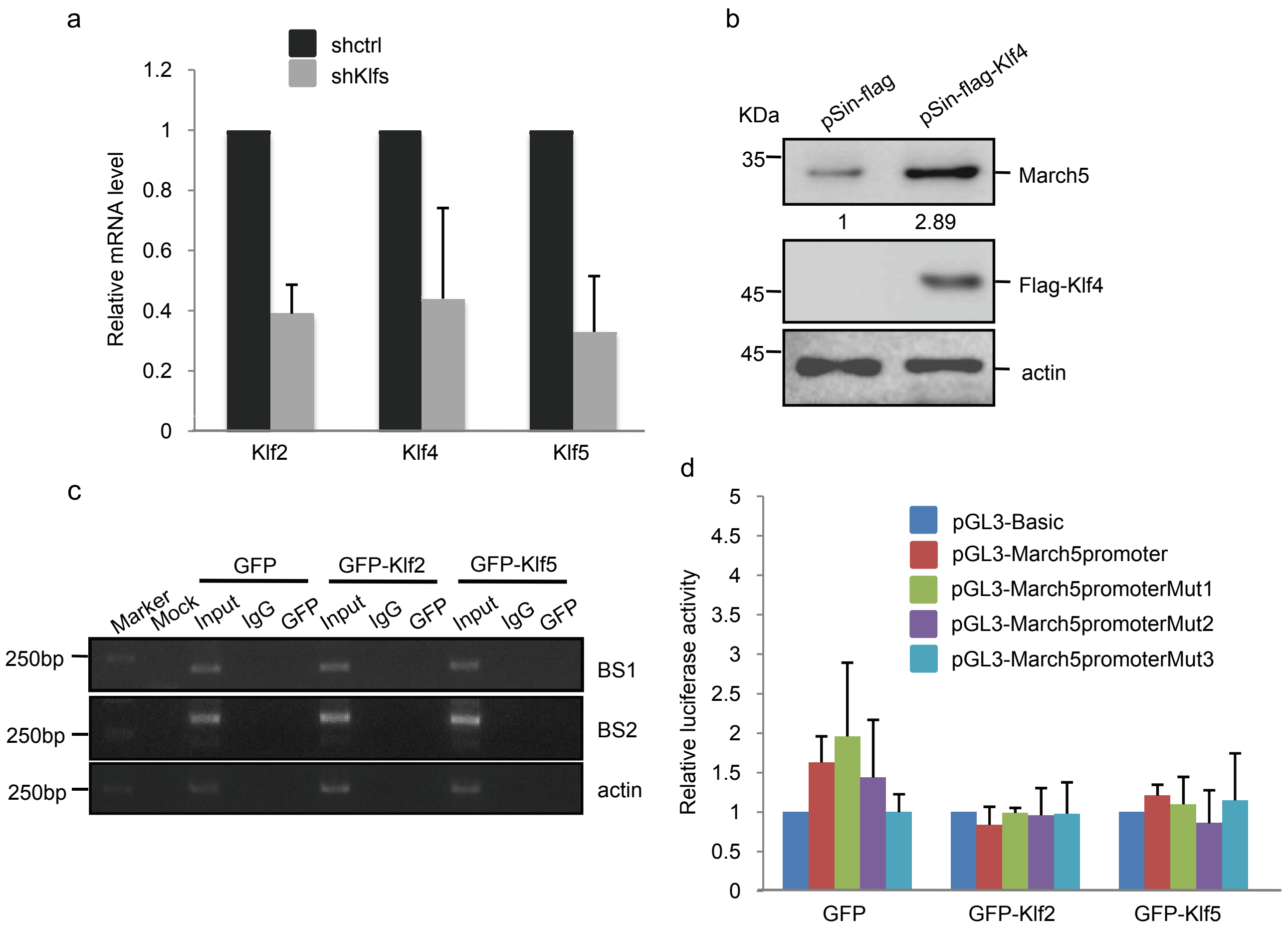

(a) E14 cells were infected with lentiviruses expressing the indicated shRNAs. Twenty-four hours after infection, total RNA was extracted from these cells before real-time RT-PCR was performed. Data are shown as mean ± SD from three independent experiments.

(b) E14 cells were infected with lentiviruses expressing either Flag-Klf4 or control proteins. Seventy-two hours later, cell lysates were analyzed by Western blot. The value of each band indicates the relative expression level after normalizing to the loading control actin.

(c) MEF cells were transfected with GFP, GFP-klf2 or GFP-klf5, followed by ChIP assay using anti-GFP antibody or an isotype-matched IgG. Actin was used as a negative control.

(d) HEK 293T cells were cotransfected with the reporter plasmids in combination with the expression vectors as indicated. Renilla vector pRL-CMV was also introduced as a transfection internal control. Twenty-four hours later, reporter activity was measured and plotted after normalizing with respect to Renilla luciferase activity. Data are shown as mean ± SD of three independent experiments.

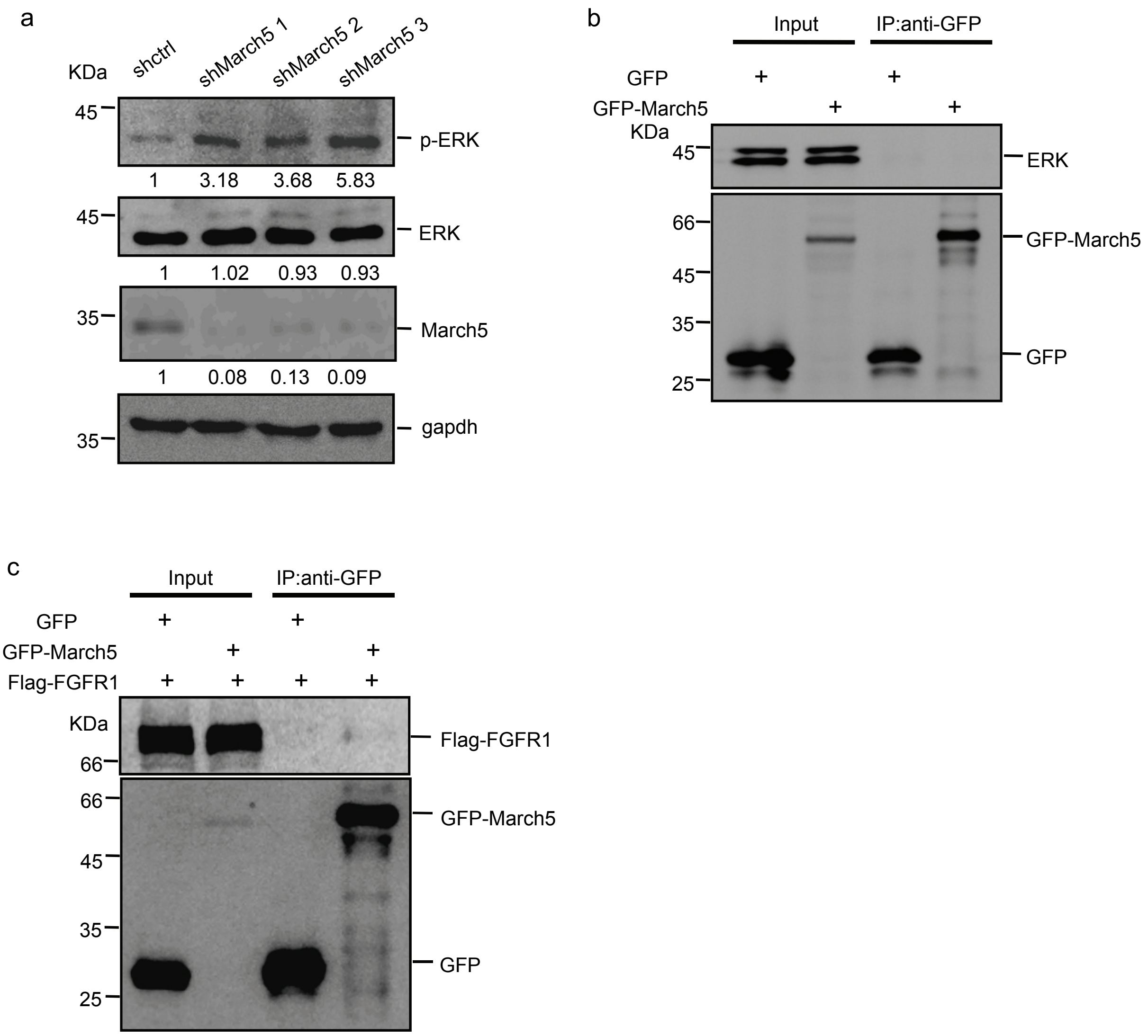

(a) E14 cells were infected with lentiviruses expressing three different March5 shRNAs or control shRNA. Cells were maintained in mESCs culture medium in the presence of LIF as indicated for 4 days. Cell lysates were then analyzed by Western blot with the indicated antibodies. The value of each band indicates the relative expression level after normalizing to the loading control gapdh

(b) HEK 293T cells were transfected with either GFP-March5 or control vector. Twenty-four hours later, cell lysates were immunoprecipitated with anti-GFP antibody, followed by Western blot analysis with anti-ERK antibody.

(c) HEK 293T cells were transfected with GFP-March5 and Flag-FGFR1 as indicated. Twenty-four hours later, cell lysates were immunoprecipitated with anti-GFP antibody, followed by Western blot analysis with the indicated antibodies.

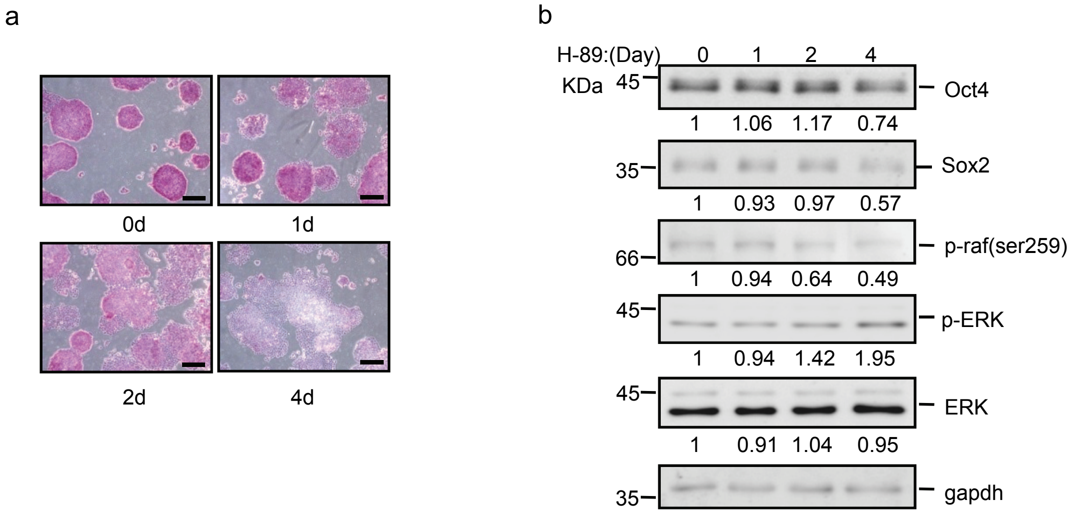

(a) E14 cells were treated by H-89 for indicated periods of time before they were stained for alkaline phosphotase activity. Shown images are representative of three independent experiments. Scale bar represents 100μm.
(b) E14 cells were treated by H-89 for indicated periods of time. Cell lysates were analyzed by Western blot with the indicated antibodies. The value of each band indicates the relative expression level after normalizing to the loading control gapdh.

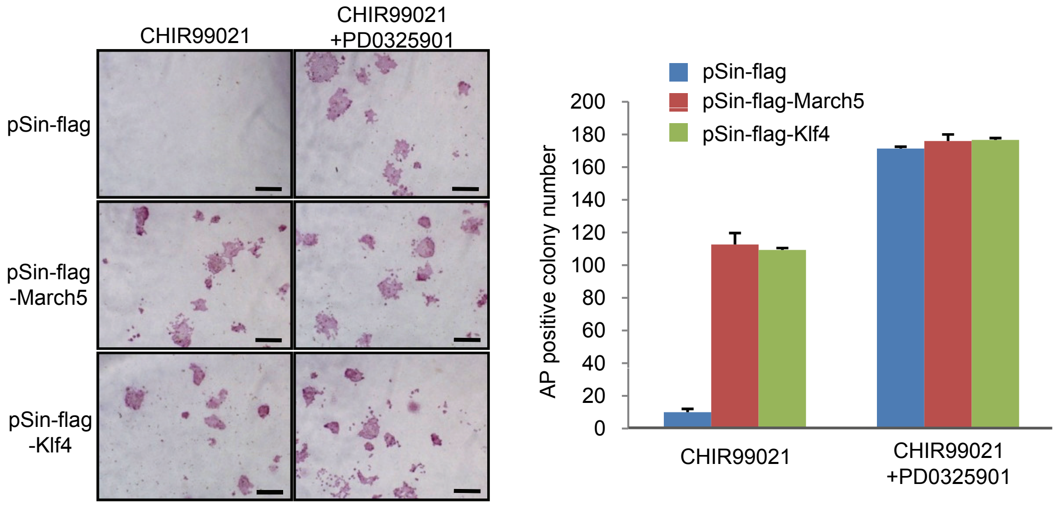

R1 ES cells expressing Flag-March5, Flag-Klf4 or control proteins were plated on matrigel-coated dish at clonal density in mESC culture medium for 1 day. The culture medium was then changed to N2B27 medium supplemented with either GSK3β inhibitor CHIR99021 alone or GSK3β inhibitor CHIR99021 and MEK/ERK inhibitor PD0325901. After 3 passages, the ES cell colonies were stained for alkaline phosphotase activity. The shown images are representative from three independent experiments. The number of AP-positive colonies was also counted and shown as mean ± SD from three independent experiments.

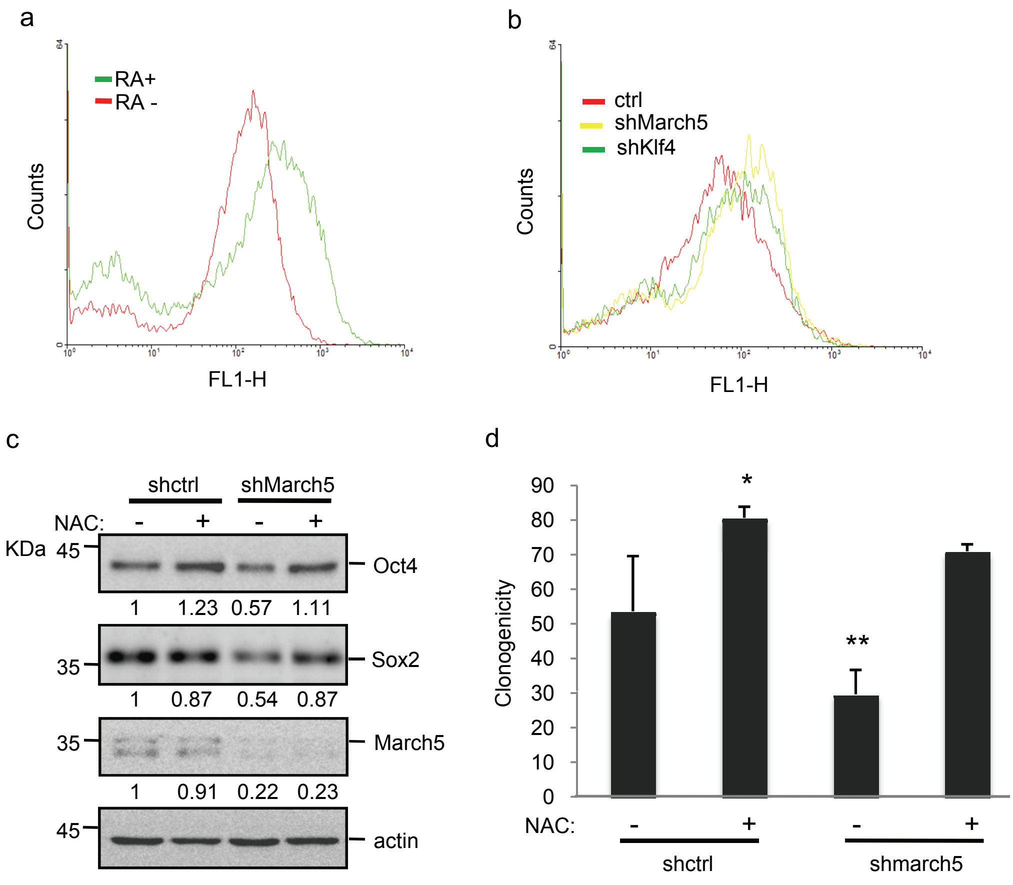

(a) E14 cells were treated with 10^{-7}M RA for 4 days. Mitochondrial ROS production was then measured by staining cells with dichlorodihydrofluorescien, followed by FACS analysis.

(b) ROS production in E14 cells expressing control, March5 or Klf4 specific shRNAs was measured as described in (a).

(c and d) E14 cells expressing either control or March5 specific shRNA were treated with ROS scavenger NAC (2μM) for 4 days. (c) Expression of pluripotency genes was determined by western blot. The value of each band indicates the relative expression level after normalizing to the loading control actin. (d) The forming colonies were stained for alkaline phosphotase activity, and AP-positive colonies were also counted. Data are shown as mean ± SD of three independent experiments. * and ** indicates $P < 0.05$ and $P < 0.01$, respectively.

Supplementary Figure 8

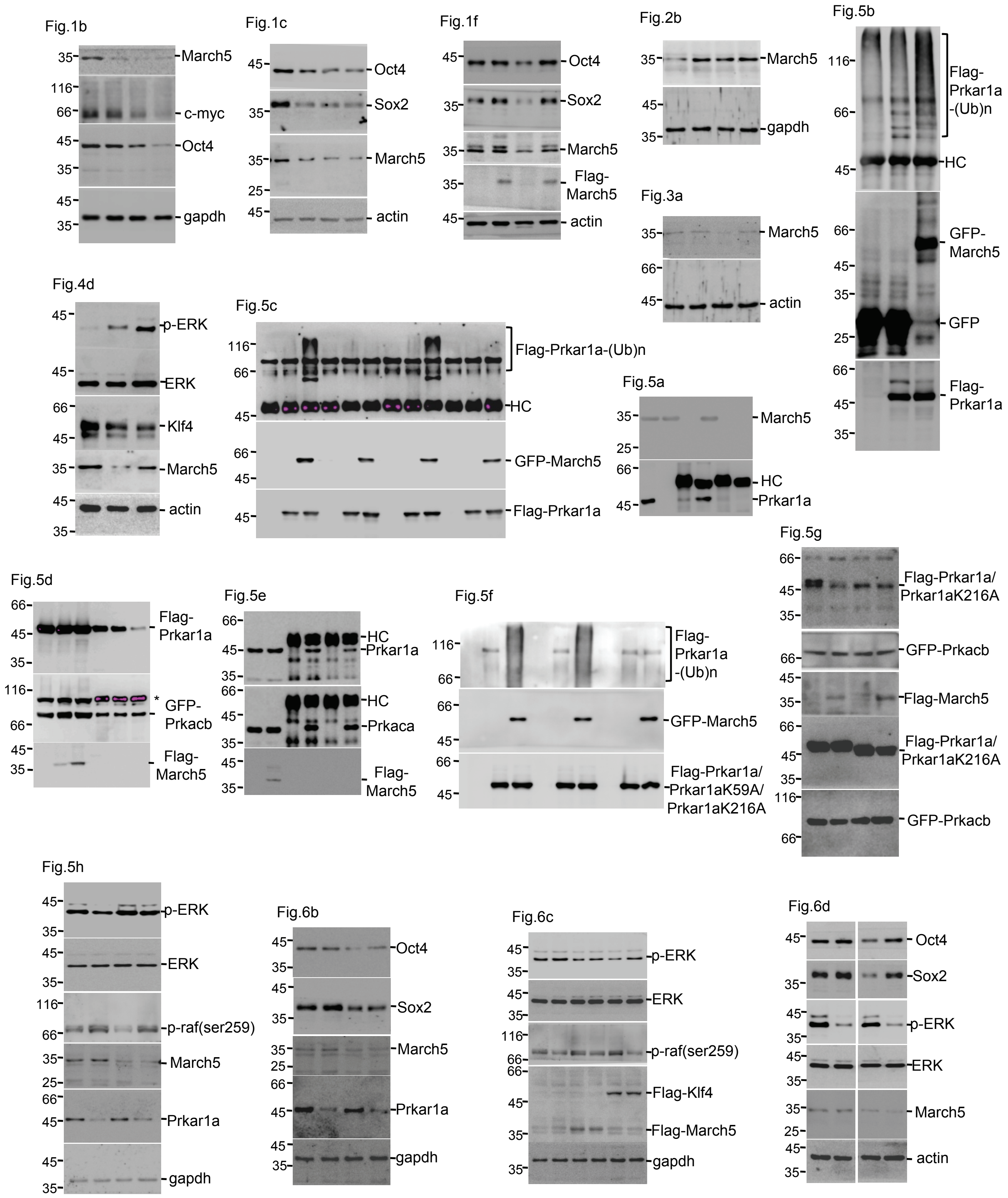

Supplementary Fig. 8: Uncropped scans of immunoblots.

Supplementary Figure 9

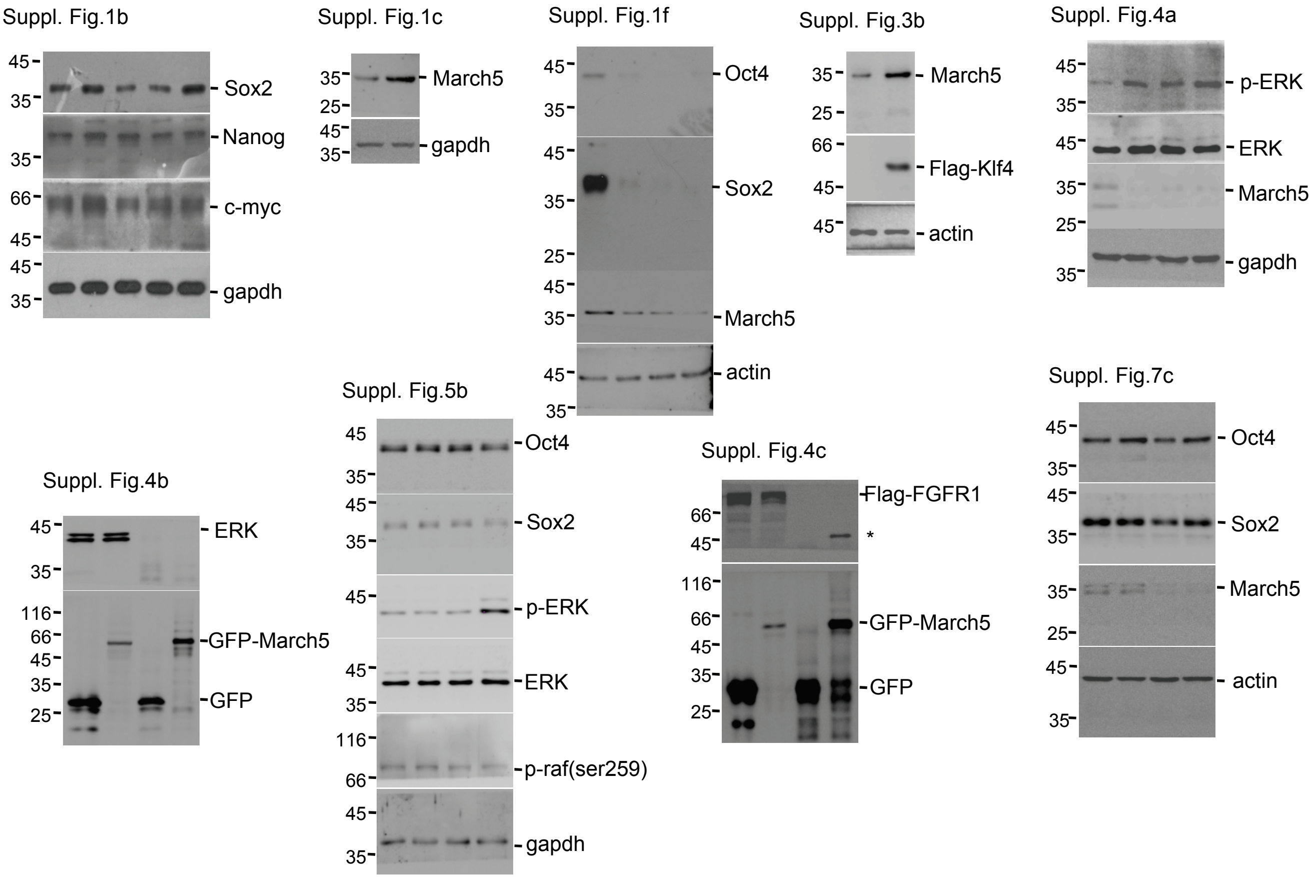

Supplementary Fig. 9: Uncropped scans of immunoblots.

Supplementary Table 1. List of genes identified by primary screen

Mus musculus suppressor of Ty 5 homolog (S. cerevisiae) (Supt5h), mRNA
Mus musculus blocked early in transport 1 homolog (S. cerevisiae)-like (Bet1l), mRNA
Mus musculus G protein-coupled receptor 133 (Gpr133), mRNA
Mus musculus ethanolamine kinase 1 (Etnk1), mRNA
Mus musculus CCAAT/enhancer binding protein zeta (Cebpz), mRNA
Mus musculus membrane-associated ring finger (C3HC4) 5 (March5), transcript variant 1, mRNA
Mus musculus high mobility group box 2 (Hmgb2), mRNA
PREDICTED: Mus musculus high mobility group protein B2-like (LOC100041490), miscRNA
PREDICTED: Mus musculus predicted gene 13237 (Gm13237), miscRNA
PREDICTED: Mus musculus predicted gene 13167 (Gm13167), miscRNA
PREDICTED: Mus musculus predicted gene 13232 (Gm13232), miscRNA
PREDICTED: Mus musculus predicted gene 13160 (Gm13160), miscRNA
Mus musculus ribosomal protein L27 (Rpl27), mRNA
PREDICTED: Mus musculus predicted pseudogene 4825 (Gm4825), mRNA
Mus musculus ATP-binding cassette, sub-family F (GCN20), member 1 (Abcf1), mRNA
Mus musculus Ttk protein kinase (Ttk), transcript variant 1, mRNA
Mus musculus heat shock protein 90, alpha (cytosolic), class A member 1 (Hsp90aa1), mRNA
Mus musculus ribosomal protein S8 (Rps8), mRNA
PREDICTED: Mus musculus predicted pseudogene 15501, transcript variant 1 (Gm15501), mRNA
PREDICTED: Mus musculus 40S ribosomal protein S8-like (LOC100862183), mRNA
Mus musculus RIKEN cDNA 2410003K15 gene (2410003K15Rik), mRNA
PREDICTED: Mus musculus RIKEN cDNA 6030426L16 gene (6030426L16Rik), miscRNA
PREDICTED: Mus musculus predicted gene 4983 (Gm4983), miscRNA
PREDICTED: Mus musculus hippocalcin-like protein 1-like (LOC385063), miscRNA

Supplementary Table 2. List of March5-interacting candidate proteins from MS analysis

Scan(s)	Peptide	z	XC	DeltaCn
MFN2 Mitofusin-2				
	K.AGWLDSELNMFTHQYLQPSR.J	2	3.3584	0.4857
	K.AGWLDSELNMFTHQYLQPSR.J	2	3.71	0.3868
	K.AQGMPEGGGALAEGFQVR.M	2	3.1666	0.4828
	K.AQGMPEGGGALAEGFQVR.M	2	2.4978	0.4493
	K.AQGMPEGGGALAEGFQVR.M	2	4.5184	0.5572
	K.AQGMPEGGGALAEGFQVR.M	2	4.6773	0.622
	K.AQGMPEGGGALAEGFQVR.M	2	3.4531	0.5899
	K.AQGMPEGGGALAEGFQVR.M	2	2.8166	0.4519
	K.FIDKQLELLAQDYK.L	2	4.5172	0.5037
	K.INGIFEQLGAYIQESATFLEDTYR.N	2	3.3948	0.3355
	R.CSTAITNSLQTMQQDMIDGLKPLLPVSVR.S	3	3.8533	0.5186
	R.CTSFLVDELGVVDR.S	2	4.334	0.5022
	R.CTSFLVDELGVVDR.S	2	5.0839	0.5789
	R.ENLEQEIAAMNK.K	2	2.6478	0.3695
	R.ENLEQEIAAMNK.K	2	2.8446	0.4711
	R.ENLEQEIAAMNK.K	2	3.6318	0.4341
	R.ENLEQEIAAMNK.K	2	2.8073	0.422
	R.LSRPNIFILNNR.W	2	3.2588	0.2875
	R.LSVLVDDYQMDFHPSPVVLK.V	2	4.7718	0.4057
	R.MFEFQNFER.R	2	3.0914	0.4728
	R.MFEFQNFER.R	2	3.3476	0.5194
	R.MFEFQNFER.R	2	2.9807	0.503
	R.MFEFQNFER.R	2	2.9342	0.5182
	R.NAELDPVTTEEQVLDVK.G	2	5.6525	0.5857
	R.NAELDPVTTEEQVLDVK.G	2	5.5153	0.6165
	R.NAELDPVTTEEQVLDVK.G	2	4.8286	0.4978
	R.NAELDPVTTEEQVLDVK.G	2	2.4442	0.4232
MARCH5 E3 ubiquitin-protein ligase MARCH5				
	K.LGPVVYVLDLADR.L	2	4.5655	0.6201
	K.LGPVVYVLDLADR.L	2	4.7952	0.6238
	K.LGPVVYVLDLADR.L	2	4.5112	0.5717
	K.LGPVVYVLDLADR.L	2	4.2321	0.5958
	K.LMFSSVNSNLQR.T	2	2.7108	0.4466
	K.LQILNSIFPGIGCPVPR.I	2	2.54	0.2286
	K.LQILNSIFPGIGCPVPR.I	2	3.0611	0.3731
	K.LQILNSIFPGIGCPVPR.I	2	3.8192	0.4184
	K.LQILNSIFPGIGCPVPR.I	2	4.1082	0.5876
	K.LQILNSIFPGIGCPVPR.I	2	4.3389	0.5555
	R.ADPLFLLIGLPTIPVMLILGK.M	2	2.8316	0.3773

	R.ILCGALVFPTIATIVGK.L	2	3.6239	0.4508
	R.ILCGALVFPTIATIVGK.L	2	3.9308	0.5068
	R.SCWVCFATDEDDR.T	2	3.7078	0.1238
	R.SCWVCFATDEDDR.T	2	4.7819	0.5151
	R.SCWVCFATDEDDR.T	2	4.3688	0.5879
	R.SCWVCFATDEDDR.T	2	4.5938	0.6228
	R.SCWVCFATDEDDR.T	2	3.9577	0.4892
	R.SCWVCFATDEDDR.T	2	3.6101	0.4386
	R.SCWVCFATDEDDR.T	2	3.0841	0.2382
	R.SCWVCFATDEDDRTAEWVRPCR.C	3	5.1743	0.5224
	R.SCWVCFATDEDDRTAEWVRPCR.C	3	4.5194	0.4209
	R.SCWVCFATDEDDRTAEWVRPCR.C	3	4.9349	0.4262
	R.SCWVCFATDEDDRTAEWVRPCR.C	3	4.8232	0.4606
	R.VACPQCNAEYLIVFPK.L	2	3.4529	0.3911
	R.VACPQCNAEYLIVFPK.L	2	3.6355	0.3701
	R.VACPQCNAEYLIVFPK.L	2	4.5241	0.5225
	R.VACPQCNAEYLIVFPK.L	2	4.3935	0.4539
	R.VACPQCNAEYLIVFPK.L	2	4.1787	0.4342
	R.VACPQCNAEYLIVFPK.L	2	3.0097	0.3398
	R.VACPQCNAEYLIVFPK.L	2	3.1175	0.4284
	R.VACPQCNAEYLIVFPK.L	2	2.7713	0.2672
	R.VACPQCNAEYLIVFPK.L	2	2.2828	0.3565
UBAC2 Ubiquitin-associated domain-containing protein 2				
	R.ASNNDLNVATNFLLQH.-	2	4.2371	0.4739
	R.ASNNDLNVATNFLLQH.-	2	4.0601	0.487
	R.QLMFSQFAQGR.R	2	2.6275	0.2776
	R.QLMFSQFAQGR.R	2	2.2573	0.294
MIA3 Melanoma inhibitory activity protein 3				
	K.EQLQQEIEDWSK.L	2	2.6658	0.4574
	R.TQTAISVVEEDLK.L	2	3.8173	0.447
HS2ST1 Heparan sulfate 2-O-sulfotransferase 1				
	K.TASTSFTNIAYDLCAK.N	2	2.2675	0.2062
	R.EKDGDLYILAQNFFYEK.I	2	2.2508	0.2221
BAG2 BAG family molecular chaperone regulator 2				
	R.LLESLDQLELR.V	2	3.1592	0.4252
	R.LLESLDQLELR.V	2	3.7391	0.4283
S100A9 Protein S100-A9				
	R.NIETIINTFHQYSVK.L	2	4.4622	0.5003
	R.NIETIINTFHQYSVK.L	2	4.0824	0.441
RLIM E3 ubiquitin-protein ligase RLIM				
	R.ILNTGLSETTSVAIQTMLR.Q	2	2.8402	0.4362
	R.ILNTGLSETTSVAIQTMLR.Q	2	3.2741	0.3847
ZNF564 Zinc finger protein 564				

	K.WEDQSIEDWYKNQGRILR.N	2	2.6282	0.1546
	K.WEDQSIEDWYKNQGRILR.N	2	2.5351	0.2304
FAF2 FAS-associated factor 2				
	K.LLQFQDLTGIESMDQCR.H	2	4.3754	0.573
	K.LLQFQDLTGIESMDQCR.H	2	4.6092	0.6091
OLFML2A Olfactomedin-like 2A				
	R.SNSAEPNSAEQDEAEPR.S	2	2.6145	0.1677
	R.SNSAEPNSAEQDEAEPR.S	2	2.5693	0.1296
ITGA3 Isoform Alpha-3B of Integrin alpha-3				
	R.QQRYLLLAGAPRELAVPDGYTNR.T	2	2.3996	0.1588
	R.QQRYLLLAGAPRELAVPDGYTNR.T	2	2.34	0.1743
SDCBP Syntenin-1				
	R.LYPELSQYMGLSLNEEEIR.A	2	2.6906	0.3577
	R.LYPELSQYMGLSLNEEEIR.A	2	3.5406	0.3833
ZCCHC4 Zinc finger CCHC domain-containing protein 4				
	R.SCQFLVDLLSALGFR.R	2	2.3526	0.2763
	R.SCQFLVDLLSALGFR.R	2	2.3164	0.1469
DDB2 Isoform D3 of DNA damage-binding protein 2				
	R.YNLIVVGRYPDPNFK.S	2	3.0418	0.2014
	R.YNLIVVGRYPDPNFK.S	2	2.491	0.1031
BAT3 cDNA FLJ60983, highly similar to Large proline-rich protein BAT3				
	R.MATHTLITGLEEYVR.E	2	2.7038	0.304
	R.MATHTLITGLEEYVR.E	2	2.7086	0.3264
ATP1A3 cDNA FLJ59513, highly similar to Sodium/potassium-transporting ATPase alpha-3 chain				
	K.GVGIISEGNETVEDIAAR.L	2	2.3873	0.1212
	K.GVGIISEGNETVEDIAAR.L	2	2.2185	0.2265
TECPR1 Tectonin beta-propeller repeat-containing protein 1				
	R.DVSIIPESPGAEGSGHSIALWAVSDK.G	2	2.4872	0.178
	R.DVSIIPESPGAEGSGHSIALWAVSDK.G	2	2.661	0.2118
EXT2 Exostosin-2				
	K.M*SDVYSILQSTPQR.Q	2	2.2004	0.2683
	K.M*SDVYSILQSTPQR.Q	2	2.4481	0.3729
NPLOC4 Nuclear protein localization protein 4 homolog				
	K.VFGAPNVVEDEIDQYLSK.Q	2	4.1496	0.6364
UBL4A Ubiquitin-like protein 4A				
	R.LADSPPPQVWQLISK.V	2	2.3558	0.1891
SERPINF1 Pigment epithelium-derived factor				
	K.ELLDTVTAHQKNLK.J	2	2.8104	0.1109
PEX3 Peroxisomal biogenesis factor 3				
	K.KCIFLGTVLGGVYILGK.Y	2	2.4951	0.1522
CAPN1 Calpain-1 catalytic subunit				
	K.GFSLESCRSMVNLM*DR.D	2	2.5165	0.1267
ENPEP Glutamyl aminopeptidase				

	R.YGMQNSGNEISWNYTLEQYQK.T	2	2.8164	0.1916
APBA2 Amyloid beta A4 precursor protein-binding family A member 2				
	R.IIEINGQSVVATAHEK.I	2	2.3289	0.1101
SDAD1 Protein SDA1 homolog				
	R.FEVKMMLMNLISR.L	2	2.7119	0.1337
PRKAR1A cAMP-dependent protein kinase type I-alpha regulatory subunit				
	R.LTVADALEPVQFEDGQK.I	2	2.544	0.1724
ESF1				
	R.FIPDDVTFDDEPK.D	2	2.7498	0.3169
JSRP1 Junctional sarcoplasmic reticulum protein 1				
	R.EAAENDEEEPGEATGEAVR.E	2	3.1568	0.1157
TNIP2 TNFAIP3-interacting protein 2				
	R.GLQIPHEPELMRKEISR.L	2	2.2748	0.255
ITCH E3 ubiquitin-protein ligase Itchy homolog				
	R.VSGNNSPSLSNGGFKPSRPPR.P	2	2.83	0.2775
ARHGAP15 Rho GTPase-activating protein 15				
	K.SMILTDVGKVTEPISR.H	2	2.4117	0.1024
DOCK7 Dedicator of cytokinesis protein 7				
	R.SIIGSKGLDRSNSWVNTGGPK.A	2	2.8012	0.1461
ANKRD13A Ankyrin repeat domain-containing protein 13A				
	R.DYHNTSMALEGVPELLQK.I	2	2.5367	0.1576
CNGA1 cGMP-gated cation channel alpha-1				
	K.LRAEIAINVHLDTLKK.V	2	2.6855	0.1045
FAM186A Protein FAM186A				
	K.SEQSNLEEFQEAIM*AFLKQK.I	2	2.2194	0.17
PALM3 Paralemmin-3				
	R.EGGEEPLGIERKVEGHLR.A	2	2.5499	0.1332
IP6K3 Inositol hexakisphosphate kinase 3				
	R.KCAQSTSACLGVRICGM*QVYQTDK.K	2	2.4292	0.244
FLRT1 fibronectin leucine rich transmembrane protein 1				
	R.RLVLDGNLLANQRIADDTFSR.L	2	2.4426	0.1122
SERPINA7 Thyroxine-binding globulin				
	K.HLKPLAKFLNDVK.T	2	2.3944	0.1389
GRIA3 Isoform Flop of Glutamate receptor 3				
	R.FAVQLYNTNQNTTEK.P	2	2.3534	0.1482
LTF cDNA FLJ78440, highly similar to Human lactoferrin				
	R.IDSGLYLGSGYFTAIQNLR.K	2	2.2399	0.2889
COMT Isoform Soluble of Catechol O-methyltransferase				
	R.YLPDTLLLEECGLLR.K	2	3.1885	0.3102
GON4L Isoform 1 of GON-4-like protein				
	R.KSTQPDVCASPQEKPLR.T	2	2.2158	0.1435
FGFR1 Basic fibroblast growth factor receptor 1				
	R.LSSSGTPMLAGVSEYELPEDPR.W	2	2.407	0.1721

USP6 Ubiquitin carboxyl-terminal hydrolase 6				
	K.ENGAGQICELADALSR.G	2	2.3919	0.2377
DAB1 Isoform DAB553 of Disabled homolog 1				
	K.QCEQAVYQVPTSQKK.E	2	2.3093	0.2582
RABGAP1L RAB GTPase-activating protein 1-like				
	K.TATGTQPLQPAPVTQPPK.E	2	2.5941	0.1684
RNF219 RING finger protein 219				
	R.SDKYIEELESQVAQLK.N	2	2.2504	0.3334
ARHGEF4 Isoform 4 of Rho guanine nucleotide exchange factor 4				
	K.MIDISLDGFLLTPVQK.I	2	2.7796	0.1322
PACRGL PACRG-like protein				
	R.LQRECPPESLSFDPLLITLAEGLR.-	2	2.6068	0.1188
ODF2L outer dense fiber of sperm tails 2-like isoform b 1				
	R.MNKNEAIVMKEASR.Q	2	2.2956	0.2241
HTRA3 Probable serine protease HTRA3				
	R.QLQKGACPLGLHQLSSPR.Y	2	2.2295	0.2665
BEND6 Chromosome 6 open reading frame 65				
	R.QSLVMLQGNKLDITTEKTILHGGMK.T	2	2.7892	0.1047
TTC25 Tetratricopeptide repeat protein 25				
	R.REPEELGKTQFGEIGETK.K	2	2.6297	0.1264
EPS15 Putative uncharacterized protein EPS15				
	K.AKYDAIFDSLSPVNGFLSGDKVK.P	2	3.0782	0.1897
FILIP1 Filamin-A-interacting protein 1				
	R.DKIAKGECGNSSLMAEVENLR.K	2	2.9235	0.2101
RTTN Rotatin				
	R.VAANALMSLLAVSRRAQK.H	2	2.2705	0.1121
CCDC88C Protein Daple				
	K.SFVFELNECASSRILKLEK.E	2	2.3707	0.2668
FSCN1 Conserved hypothetical protein				
	R.KEGAEM*HPLGR.V	2	2.4617	0.1326
ENO1 Isoform MBP-1 of Alpha-enolase				
	R.EAMRIGAEVYHNLKNVIK.E	2	2.855	0.2281
IGSF9B Putative uncharacterized protein (Fragment)				
	R.EEPEFVTARAGESVVLR.C	2	2.2167	0.1287
PARP14 82 kDa protein				
	K.EMVILAGKSEDVQSIEVQVR.E	2	2.2888	0.1182
MED1 Mediator of RNA polymerase II transcription subunit 1				
	K.EDSPGLLQFEVCPLSESR.F	2	2.3188	0.1312
LMNB1 LMNB1 protein				
	R.MRIESLSSQLSNLQK.E	2	2.8147	0.3518
SMARCE1 Putative uncharacterized protein SMARCE1				
	R.KFLESTDSFNNELK.R	2	2.6422	0.1897
FAM186B 59 kDa protein				

	R.RLQSLRQEAINHVQIMK.E	2	2.2835	0.2742
RPAP1 RNA polymerase II-associated protein 1				
	K.TSASSSYLLGALRGLR.E	2	2.9873	0.2216
SHANK3 SH3 and multiple ankyrin repeat domains protein 3				
	K.GEKLDEM*LAAAAEPTLRPDIADADSR.A	2	2.4833	0.1063
CXorf30 Putative uncharacterized protein CXorf30				
	R.ITTRIGLQSTIVIPFK.N	2	2.4187	0.1191
TYMP Putative uncharacterized protein TYMP				
	R.GFVAAVVNGSAQGAQIGAMLMAIR.L	2	3.3344	0.2343
DGCR14 Putative uncharacterized protein DGCR14				
	-.METPGASASSLLLPAASRPPR.K	2	2.2637	0.1306
HMCN1 Hemicentin-1				
	R.PCEGNAVEIIMCNIR.P	2	2.2372	0.1023
IL22RA2 Putative uncharacterized protein IL22RA2				
	R.NFHNILQWQPGR.A	2	2.3943	0.1229
UFD1L Putative uncharacterized protein UFD1L				
	K.FQPQSPDFLDITNPK.A	2	2.541	0.1798
MAPK1 Extracellular signal-regulated kinase-2 splice variant				
	K.HYLDQLNHILALDLLDK.M	2	2.2279	0.1015
PRKD3 Protein				
	R.SVVGTPAYLAPEVLRSK.G	2	2.8672	0.1263
USP34 Putative uncharacterized protein USP34				
	K.IKSAHKLLYALEIIEALGK.P	2	2.5887	0.2379
MCF2L MCF.2 cell line derived transforming sequence-like				
	R.GCTFLRGDSPHPPTLYVIECCH.-	2	2.2174	0.1846
CAB39L Calcium binding protein 39-like				
	-.MPLFSKSHKNPAEIVK.I	2	2.209	0.1448
AZGP1 Putative uncharacterized protein AZGP1				
	K.QDSQLQKAREDIFM*ETLK.D	2	2.264	0.1463
MC3R melanocortin 3 receptor				
	R.IAALPPADGVAPQQHSCMK.G	2	2.2711	0.1539
QPCTL glutaminyl-peptide cyclotransferase-like				
	R.ELRVPLIGSLPEARLR.R	2	2.5191	0.2591
CRB1 Putative uncharacterized protein CRB1				
	K.IDWNHITLENISSGSSLNVK.A	2	2.7385	0.1257
PCNXL2 Putative uncharacterized protein PCNXL2				
	K.DISESFLLDFFM*VSILFSK.L	2	2.2251	0.1423
ACTG1 Actin, cytoplasmic 2				
	K.LCYVALDFEQEMATAASSSSLEK.S	2	5.8276	0.5497
	K.LCYVALDFEQEMATAASSSSLEK.S	2	5.8035	0.6328
	K.LCYVALDFEQEMATAASSSSLEK.S	3	3.8183	0.4599
RGS14 cDNA FLJ40831 fis, clone TRACH2012138, highly similar to Homo sapiens regulator of G-protein signalling 14 (RGS14)				

	K.SAAQPIGGSLNSTTDSAL.-	2	2.2236	0.1545
DMTF1 77 kDa protein				
	R.SKWLNYLNWKQSGGTEWTK.E	2	3.4127	0.2384
guanine nucleotide binding protein (G protein), beta polypeptide 2-like				
	K.LTRDETNYGIPQR.A	2	2.95	0.35
	R.LWDLTTGTTTR.R	2	2.78	0.34
	R.YWLCAATGPSIK.I	2	3.59	0.53
	R.FSPNSSNPIIVSCGWDK.L	2	3.34	0.51
leucine rich repeat (in FLII) interacting protein 2				
	K.AMVSNAQLDNEK.T	2	2.87	0.41
	K.ELKESLSEVEEK.Y	2	2.68	0.19
	R.FSAEDEALSNIAR.E	2	4.32	0.53
tripartite motif-containing 28 protein; KRAB-associated protein 1				
	K.DHQYQFLEDAVR.N	2	2.87	0.51
	K.LSPPYSSPQEFAQDVGR.M	2	2.78	0.45
	K.LSPPYSSPQEFAQDVGR.M	2	3.29	0.50
	K.LSPPYSSPQEFAQDVGR.M	2	2.89	0.48
	K.VFPGSTTEDYNLIVIER.G	2	3.32	0.48
	K.VFPGSTTEDYNLIVIER.G	2	3.24	0.39
tyrosine 3/tryptophan 5 -monooxygenase activation protein				
	K.SVTEQGAELSNEER.N	2	4.38	0.44
	R.DICNDVLSLLEK.F	2	3.61	0.23

Supplementary Table 3. List of March5-interacting candidate proteins from MS analysis

**March5-interacting candidate partners**	
Signaling transduction	
Wnt	USP34
Hippo-YAP1	MED1, CRB1
TGF- β	AZGP1, HTRA3
NF-kB	TNIP2, LTF, SMARCE1
JAK-STAT	S100A9, IL22RA2, HTRA3
PI3K-Akt	UFD1L, ITGA3, FGFR1, TECPR1, HTRA3, CAB39L
GPCR	MAPK1, ARHGAP15, PRKD3, RGS14, DOCK7, CNGA1, RABGAP1L, ARHGEF4, FAF2, USP6, GRM4, MC3R, MCF2L
ERK	S100A9, SDCBP, PRKAR1A, ITCH, TNIP2, ARHGAP15, SHANK3, MAPK1, PRKD3, RGS14, ARHGAP15 , DOCK7, CNGA1, RABGAP1L, ARHGEF4, FAF2, USP6, STK38, FGFR1, SERPINF1, EPS15, AZGP1
Others	PALM3, GRIA3, DDB2, ENO1, LMNB1
Others	
HUWE1, UBAC2, RLIM, UBL4A, USP32, RNF219, FLRT1, RPAP1, PARP14, TECPR1, IP6K3, ATP2A2, ATP1A3, EXT2 , HS2ST1, MIA3, BAG2, GAPDHS, ZNF564, OLFML2A, ZCCHC4, BAT3, CAPN1, ENPEP, APBA2, SDAD1, ESF1, JSRP1, ANKRD13A, FAM186A, SERPINA7, COMT, GON4L, DAB1, PACRGL, ODF2L, BEND6, TTC25, FILIP1, RTTN, FSCN1, IGSF9B, FAM186B, CXorf30, TYMP, DGCR14, HMCN1, CENPC1, QPCTL, PCNXL2, PEX3, POTEE, POTEF, DMTF1	

Supplementary Table 4. List of primers and shRNAs sequences

**Primers used for plasmid construction**	
3*flag-march5	Fw: 5'gcgtcgacatgccggaccaagcccttcaac3'
	Rev: 5'cgcggatccttatgcttcttcttgctctggat3'
Pmxs-march5	Fw: 5'gcggatccatgccggaccaagcccttcaac3'
	Rev: 5'ccgctcgagttatgcttcttcttgctctggat3'
Psin3*flag-march5	Fw: 5'cgacgcgtatgccggaccaagcccttcaac3'
	Rev: 5'cggactagtttatgcttcttcttgctctggat3'
Psin3*flag-march5mut	Fw: 5'cttcctccacaaacgtaacacatagtcctcccag3'
	Rev: 5'ttacgtttgtggaggaagtactcaaataaactact3'
Pgl3-march5promoter	Fw: 5'cgctcgagtgcttagaagtgtcaaggagcct3'
	Rev: 5'cccaagcttaggtaagagtcgccgcctccgt3'
Pgl3-march5promotermut1	Fw: 5'gctcgcgtcccccatgctctcc3'
	Rev: 5'gcagagcatgggggacgcgagc3'
Pgl3-march5promotermut2	Fw: 5'cctccacgccgcctcacctgct3'
	Rev: 5'agcaggtgaggcggcgtggagg
Gst-march5	Fw: 5'gcggatccttatgccggaccaagccctt3'
	Rev: 5'ccgctcgagttatgcttcttcttgctctgg3'
3*flag-PRKAR1A	Fw: 5'gcgtcgacatggcgtctggcagtatggcaac3'
	Rev: 5'gcggatcctcagacggacagggacacgaag3'
Psin3*flag-PRKAR1A	Fw: 5'gccaattgatggcgtctggcagtatggcaac3'
	Rev: 5'gcactagttcagacggacagggacacgaag3'
**Primers used for q-RT PCR and CHIP**	
March5	Fw: 5'TTCACCAGGCTTGTCTCCA3'
	Rev: 5'GCATCACTGTCACTGCTCCA3'
Klf2	Fw: 5'CTCAGCGAGCCTATCTTGCC3'
	Rev: 5'CACGTTGTTTAGGTCCTCATCC3'
Klf4	Fw: 5'GTGCCCCGACTAACCGTTG3'
	Rev: 5'GTCGTTGAACTCCTCGGTCT3'
Klf5	Fw: 5'CCGGAGACGATCTGAAACACG3'
	Rev: 5'GTTGATGCTGTAAGGTATGCCT3'
Nanog	Fw: 5'CTCATCAATGCCTGCAGTTTTTCA3'
	Rev: 5'CTCCTCAGGGCCCTTGTCAGC3'
Rex1	Fw: 5'ACGAGGTGAGTTTTCCGAAC3'
	Rev: 5'CCTCTGTCTTCTCTTGCTTC3'
endo-Oct4	Fw: 5'TCTTTCCACCAGGCCCCCGGCTC3'
	Rev: 5'TGCGGGCGGACATGGGGAGATCC3'
exo-Oct4	Fw: CCCAGTGTGGTGGTACGGGAAATC
	Rev:AGTTGCTTTCCACTCGTGCT
endo-Sox2	Fw: TAGAGCTAGACTCCGGGCGATGA
	Rev:TTGCCTTAAACAAGACCACGAAA
exo-Sox2	Fw: CCCAGTGTGGTGGTACGGGAAATC
	Rev:TCTCGGTCTCGGACAAAAGT

Esg1	Fw: ATATCCCGCCGTGGGTGAAAGTTC
	Rev:ACTCAGCCATGGACTGGAGCATCC
Fbx15	Fw: GTTGGAATCTGCTTCTACAG
	Rev:CTTCACCAAGATTTCCGATG
Actin	Fw: 5'CTGTCCCTGTATGCCTCTG3'
	Rev: 5'ATGTCACGCACGATTTCC3'
March5CHIPBS1	Fw: 5'TCTTCGGGGAGGGACAAGTGGCTTA3'
	Rev: 5'GACAGCACCAGAACCGAGAA3'
March5CHIPBS2	Fw: 5'GGTGGGACAGAGCCAGGTGA3'
	Rev: 5'CGGTGCGAACAGTCGGGTTA3'
ActinChIP	Fw: 5'TCGATATCCACGTGACATCCA3'
	Rev: 5'GCAGCATTTTTTTACCCCCTC3'
**Sequences of shRNAs**	
sh-March5#1	5'CCGGGCTTAGACTATGGCGCAAATACTCGAGTATTTGCGCCATAGTCTAAGCTTTTTG3'
sh-March5#2	5'CCGGGTGACAGTGATGCAGGTTGTACTCGAGTACAACCTGCATCACTGTCACTTTTTG3'
sh-March5#3	5'CCGGGTTTACTTCAAACAGCAGCAACTCGAGTTGCTGCTGTTTGAAGTAAACTTTTTG3'
sh-Prkar1a#1	5'CCGGCCCTTTGAAGTGCGTTAAGTTCTCGAGAACTTAACGCACTTCAAAGGGTTTTT3'
sh-Prkar1a#2	5'CCGGGCATTCCTTCGGGAATACTTTCTCGAGAAAGTATTCCCGAAGGAATGCTTTTT3'
sh-Klf2#1	5'CCGGGCAAACAGACTGCTATTTATTCTCGAGAATAAATAGCAGTCTGTTTGCTTTTTG3'
sh-Klf2#2	5'CCGGCCTAAACAACGTGTTGGACTTCTCGAGAAGTCCAACACGTTGTTTAGGTTTTTG3'
sh-Klf4#1	5'CCGGCCAGGTAAATAAGCCTGGTTTCTCGAGAAACCAGGCTTATTTACCTGGTTTTTG3'
sh-Klf4#2	5'CCGGCTGGACCTAGACTTTATCCTTCTCGAGAAGGATAAAGTCTAGGTCCAGTTTTTG3'
sh-Klf5#1	5'CCGGGCCGCTACAATTGCTTCCAAACTCGAGTTTGGAAGCAATTGTAGCGGCTTTTTG3'
sh-Klf5#2	5'CCGGCGTATCCACTTCTGCGATTATCTCGAGATAATCGCAGAAGTGGATACGTTTTTG3'
sh-Oct4#1	5'CCGGGCCGACAACAATGAGAACCTTCTCGAGAAGGTTCTCATTGTTGTCGGCTTTTT3'
sh-Oct4#2	5'CCGGCCTACAGCAGATCACTCACATCTCGAGATGTGAGTGATCTGCTGTAGGTTTTT3'
sh-Nanog#1	5'CCGGGCCAACCTGTACTATGTTTAACTCGAGTTAAACATAGTACAGGTTGGCTTTTTG3'
sh-Nanog#2	5'CCGGCCTGAGCTATAAGCAGGTTAACTCGAGTTAACCTGCTTATAGCTCAGGTTTTTG3'
